# Correction: A new archosauromorph from South America provides insights on the early diversification of tanystropheids

**DOI:** 10.1371/journal.pone.0233216

**Published:** 2020-05-11

**Authors:** Tiane M. De-Oliveira, Felipe L. Pinheiro, Átila Augusto Stock Da-Rosa, Sérgio Dias-Da-Silva, Leonardo Kerber

Fig 2 is incorrect. The scale bar should be 5 mm instead of 10 mm. The authors have provided a corrected version here. Please see the complete, correct Fig 2 caption here.

**Fig 2:**
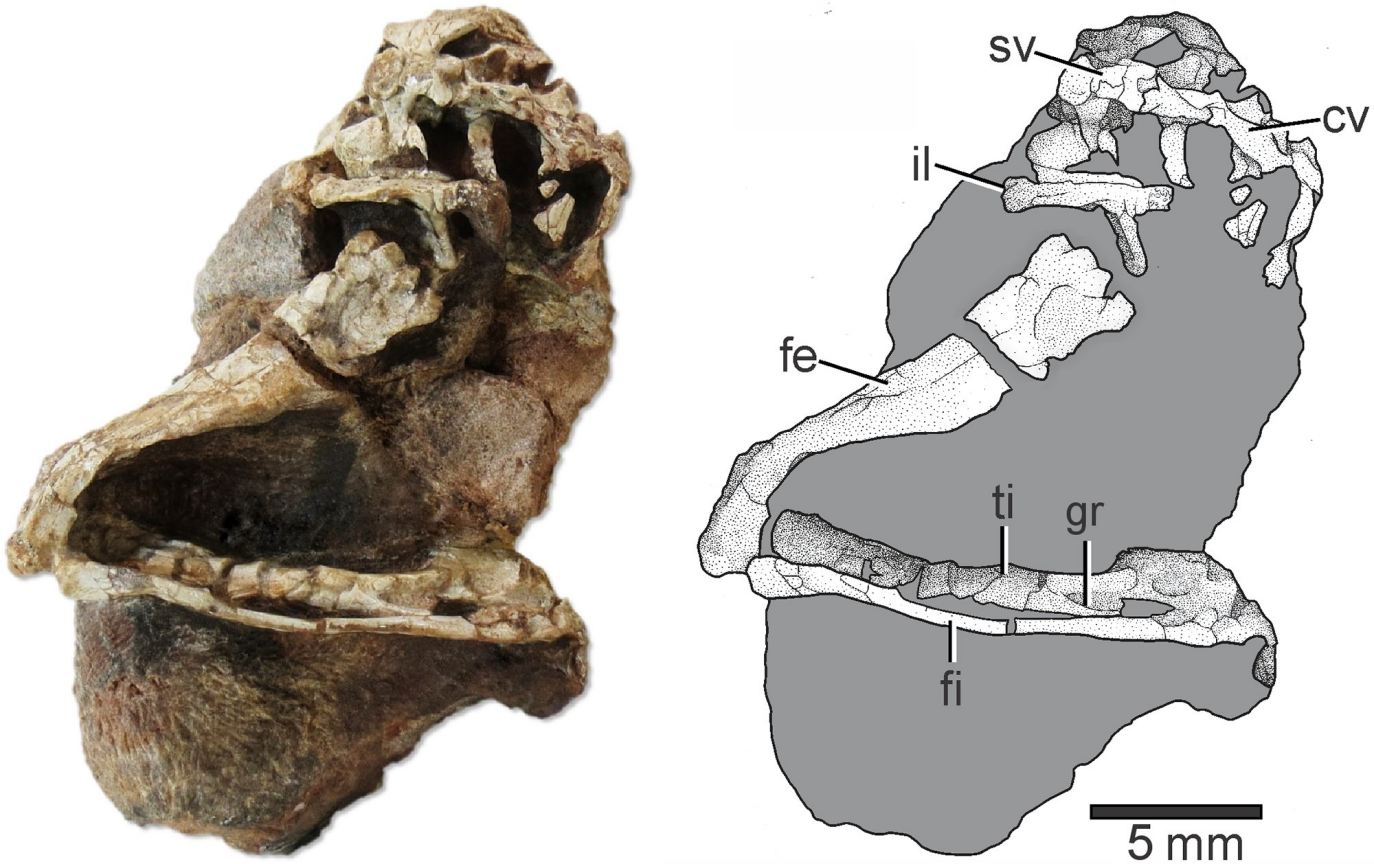
Elessaurus gondwanoccidens (UFSM 11471) from the Sanga do Cabral Formation (Lower Triassic), Brazil. Photograph and explanatory drawing respectively. Abbreviations: fe, femur; ti, tibia; gr, groove; fi, fibula; il, ilium; sv, sacral vertebra; cv, caudal vertebrae.

The captions for Figs 3 and 4 are incorrectly switched. The caption that appears with Fig 3 should appear with Fig 4, and the caption that appears with Fig 4 should appear with Fig 3. Please see the complete, correct Figs 3 and 4 captions here.

**Fig 3:**
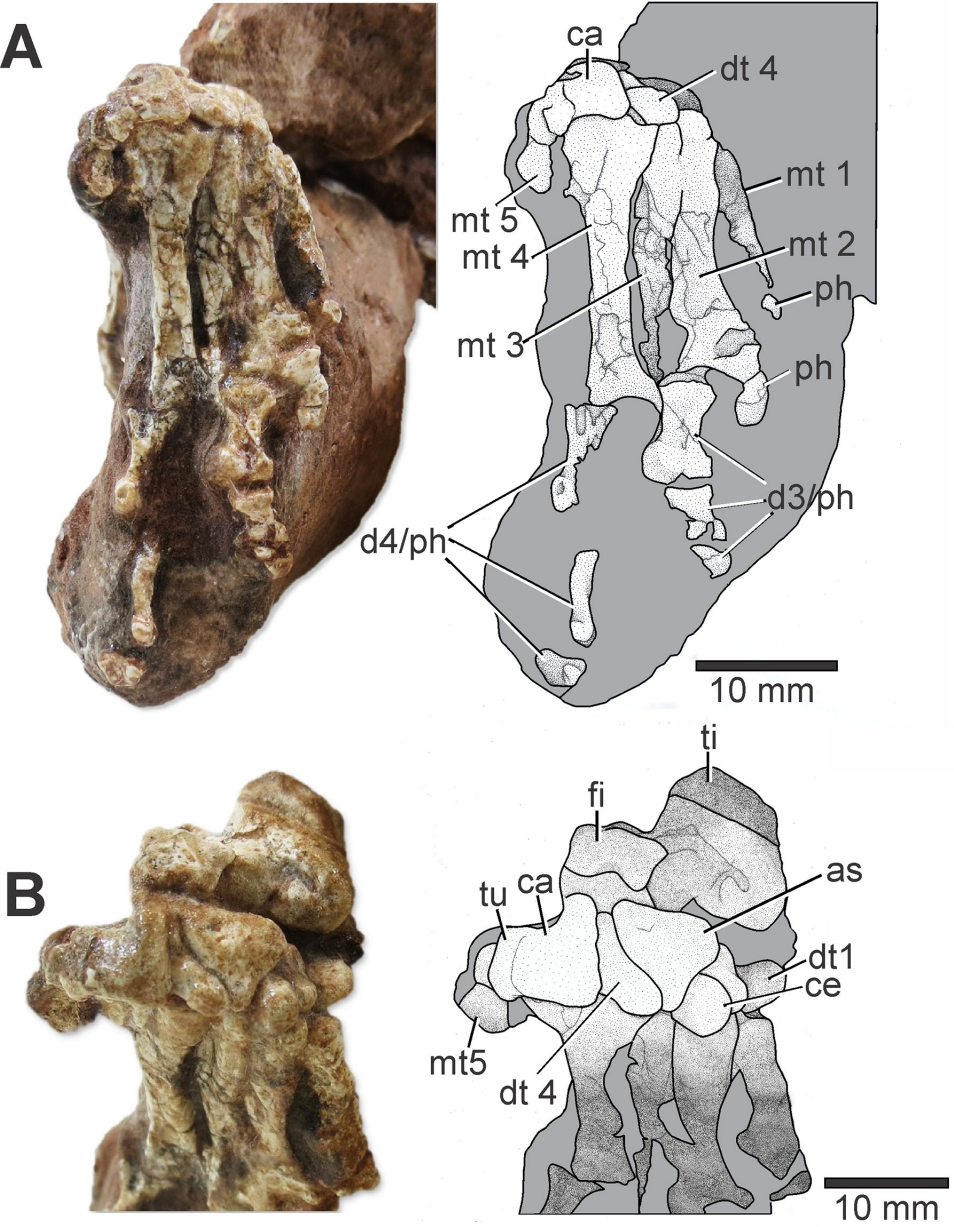
Plantar (A) and posteroplantar (B) views of the pes of Elessaurus gondwanoccidens (UFSM 11471) from the Sanga do Cabral Formation (Lower Triassic), Brazil. Photographs and explanatory drawings respectively. Abbreviations (A): ca, calcaneum; dt 4, distal tarsal 4; mt. metatarsal 1–5; d3—d4, digits; ph, phalange. (B) ti, tibia; fi, fıbula; ca, calcaneum; as. astragalus; tu, calcaneal tuber; mt, metatarsal 5; dt 1, distal tarsal 1; ce, centrale; dt 3, distal tarsal 3; dt 4, distal tarsal 4.

**Fig 4:**
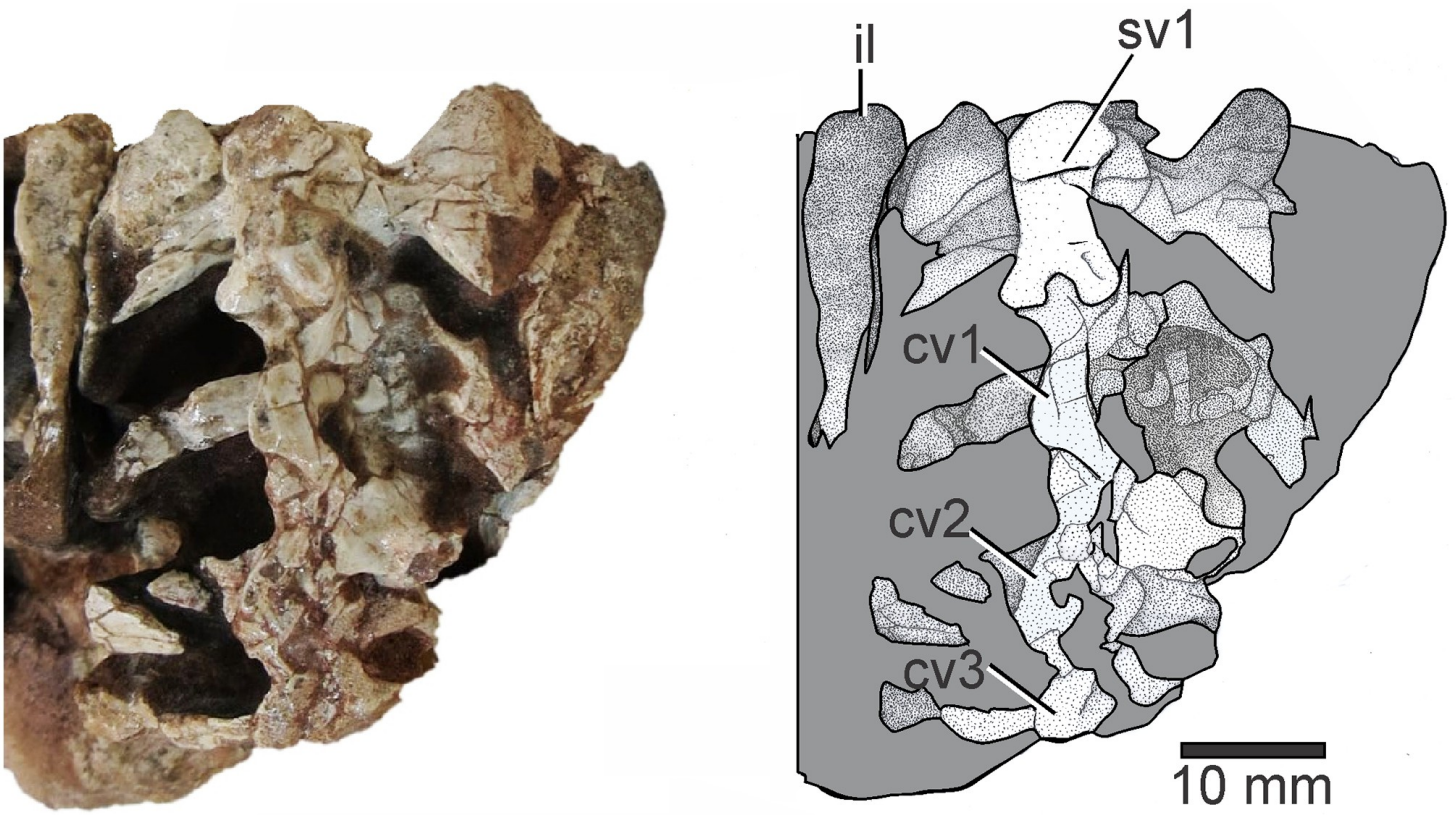
Sacral and caudal vertebrae of Elessaurus gondwanoccidens (UFSM 11471) in dorsal view. Photograph and explanatory drawing respectively. Abbreviations: sv2, second sacral vertebra; cv, caudal vertebrae 1–3.
