# Supplementary material for: A new archosauromorph from South America provides insights on the early diversification of tanystropheids
Source: PLoS One. 2020 Apr 8;15(4):e0230890. doi: 10.1371/journal.pone.0230890 (PMC7141609; doi:10.1371/journal.pone.0230890)
Supplement: S1 Fig — (DOCX) [file pone.0230890.s001.docx]

**Supplementary Material**


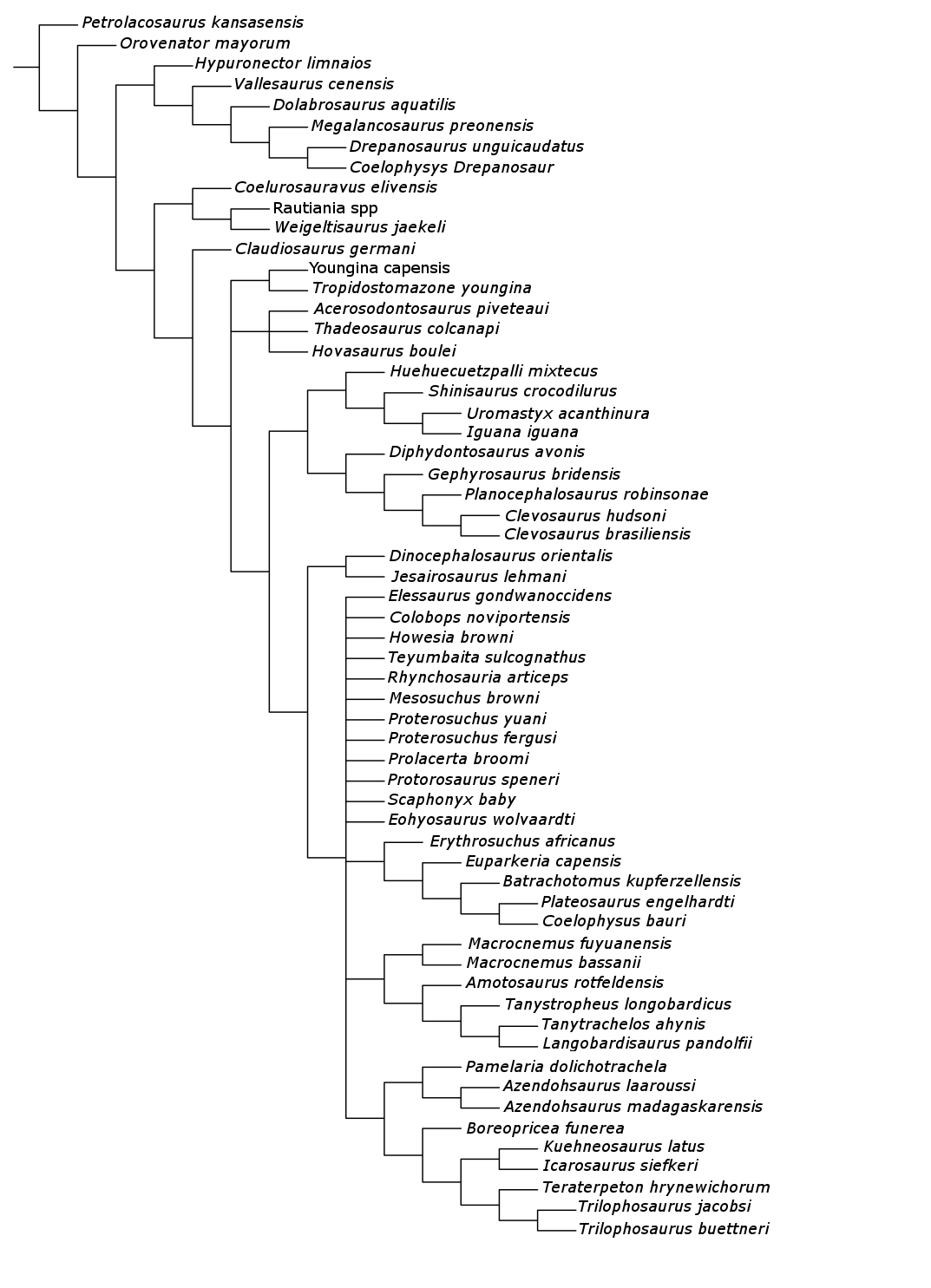


**Supplementary figure 1**. Strict consensus of the phylogenetic analysis including *Jesairosaurus lehmani* and *Dinocephalosaurus orientalis,* in the matrix of Pritchard et al. 2018.
