# Supplementary material for: A new archosauromorph from South America provides insights on the early diversification of tanystropheids
Source: PLoS One. 2020 Apr 8;15(4):e0230890. doi: 10.1371/journal.pone.0230890 (PMC7141609; doi:10.1371/journal.pone.0230890)
Supplement: Attachment — Submitted filename: PONE-D-19-19097_reviewer_revision.pdf [file pone.0230890.s003.pdf]

PLOS ONE

A NEW ARCHOSAUROMORPH FROM SOUTH AMERICA PROVIDES INSIGHTS ON THE EARLY DIVERSIFICATION OF TANYSTROPHEIDS

--Manuscript Draft--

**Manuscript Number:**	PONE-D-19-19097
**Article Type:**	Research Article
**Full Title:**	A NEW ARCHOSAUROMORPH FROM SOUTH AMERICA PROVIDES INSIGHTS ON THE EARLY DIVERSIFICATION OF TANYSTROPHEIDS
**Short Title:**	A NEW ARCHOSAUROMORPH FROM SOUTH AMERICA
**Corresponding Author:**	Tiane Macedo De-Oliveira, MS<br>Universidade Federal de Santa Maria Centro de Ciencias Naturais e Exatas<br>Santa Maria, Rio Grande do Sul BRAZIL
**Keywords:**	Diapsida; Archosauromorpha; Tanystropheidae; Triassic; Sanga do Cabral Formation; Western Gondwana; Paleobiology
**Abstract:**	After the Permo-Triassic mass extinction, the archosauromorph fossil record is comparatively abundant and ecologically diverse. Among early archosauromorphs, tanystropheids gained considerable attention due to the presence of extreme skeletal adaptations in response to sometimes overspecialized lifestyles. The origin and early radiation of Tanystropheidae, however, remains elusive. Here, a new Early Triassic archosauromorph is described and phylogenetically recovered as the sister-taxon of Tanystropheidae. The new specimen, considered a new genus and species, comprises a complete posterior limb articulated with pelvic elements and an almost complete pes. It was recovered from the Sanga do Cabral Formation (Sanga do Cabral Supersequence, Lower Triassic of Paraná Basin, Southern Brazil), which have already yielded a typical Early Triassic vertebrate assemblage of temnospondyls, procolophonoids and scarce archosauromorph remains. This new taxon provides insights on the early diversification of tanystropheids, as well as being further evidence for a premature wide geographical distribution of this clade. The morphology of the new specimen is consistent with a terrestrial lifestyle, suggesting that this condition as plesiomorphic for Tanystropheidae.
**Order of Authors:**	Tiane Macedo De-Oliveira, MS
	Felipe Lima Pinheiro
	Átila Augusto Stock Da-Rosa
	Sérgio Dias-Da-Silva
	Leonardo Kerber
**Additional Information:**	
**Question**	**Response**
**Financial Disclosure**<br><br>Enter a financial disclosure statement that describes the sources of funding for the work included in this submission. Review the submission guidelines for detailed requirements. View published research articles from *PLOS ONE* for specific examples.<br><br>This statement is required for submission and **will appear in the published article** if the submission is accepted. Please make	TMO was supported by grants from Coordenação de Aperfeiçoamento de Pessoal de Nível Superior – Brasil (CAPES) – Finance Code 001<br>FLP is supported by grants from Conselho Nacional de Desenvolvimento Científico e Tecnológico (CNPq process numbers 407969/2016-0, 305758/2017-9) and Fundação de Amparo à Pesquisa do Estado do Rio Grande do Sul (FAPERGS process number 16/2551-0000271-1).<br>SDS is supported by Conselho Nacional de Desenvolvimento Científico e Tecnológico (CNPq) (research grant, process number 306352/2016-8).<br>LK is supported by Fundação de Amparo à Pesquisa do Estado do Rio Grande do Sul (FAPERGS 17/2551-0000816-2) and Conselho Nacional de Desenvolvimento Científico e Tecnológico (CNPq 422568/2018-0).<br><br>The funders had no role in study design, data collection and analysis, decision to

Powered by Editorial Manager® and ProduXion Manager® from Aries Systems Corporation

sure it is accurate.<br><br>**Unfunded studies**<br>Enter: *The author(s) received no specific funding for this work.*<br><br>**Funded studies**<br>Enter a statement with the following details:<br>• Initials of the authors who received each award<br>• Grant numbers awarded to each author<br>• The full name of each funder<br>• URL of each funder website<br>• Did the sponsors or funders play any role in the study design, data collection and analysis, decision to publish, or preparation of the manuscript?<br>• **NO** - Include this sentence at the end of your statement: *The funders had no role in study design, data collection and analysis, decision to publish, or preparation of the manuscript.*<br>• **YES** - Specify the role(s) played.<br><br>* typeset	publish, or preparation of the manuscript.
**Competing Interests**<br><br>Use the instructions below to enter a competing interest statement for this submission. On behalf of all authors, disclose any competing interests that could be perceived to bias this work—acknowledging all financial support and any other relevant financial or non-financial competing interests.<br><br>This statement **will appear in the published article** if the submission is accepted. Please make sure it is accurate. View published research articles from *PLOS ONE* for specific examples.	The authors have declared that no competing interests exist.

**NO authors have competing interests**<br><br>Enter: *The authors have declared that no competing interests exist.*<br><br>**Authors with competing interests**<br><br>Enter competing interest details beginning with this statement:<br><br>*I have read the journal's policy and the authors of this manuscript have the following competing interests: [insert competing interests here]*<br><br>* typeset	
**Ethics Statement**<br><br>Enter an ethics statement for this submission. This statement is required if the study involved:<br><br>• Human participants<br>• Human specimens or tissue<br>• Vertebrate animals or cephalopods<br>• Vertebrate embryos or tissues<br>• Field research<br><br>Write "N/A" if the submission does not require an ethics statement.<br><br>General guidance is provided below. Consult the submission guidelines for detailed instructions. **Make sure that all information entered here is included in the Methods section of the manuscript.**	N/A

**Format for specific study types**<br><br>**Human Subject Research (involving human participants and/or tissue)**<br>• Give the name of the institutional review board or ethics committee that approved the study<br>• Include the approval number and/or a statement indicating approval of this research<br>• Indicate the form of consent obtained (written/oral) or the reason that consent was not obtained (e.g. the data were analyzed anonymously)<br><br>**Animal Research (involving vertebrate animals, embryos or tissues)**<br>• Provide the name of the Institutional Animal Care and Use Committee (IACUC) or other relevant ethics board that reviewed the study protocol, and indicate whether they approved this research or granted a formal waiver of ethical approval<br>• Include an approval number if one was obtained<br>• If the study involved *non-human primates*, add *additional details* about animal welfare and steps taken to ameliorate suffering<br>• If anesthesia, euthanasia, or any kind of animal sacrifice is part of the study, include briefly which substances and/or methods were applied<br><br>**Field Research**<br><br>Include the following details if this study involves the collection of plant, animal, or other materials from a natural setting:<br>• Field permit number<br>• Name of the institution or relevant body that granted permission	
**Data Availability**<br><br>Authors are required to make all data underlying the findings described fully available, without restriction, and from the time of publication. PLOS allows rare exceptions to address legal and ethical concerns. See the PLOS Data Policy and FAQ for detailed information.	Yes - all data are fully available without restriction

A Data Availability Statement describing where the data can be found is required at submission. Your answers to this question constitute the Data Availability Statement and **will be published in the article**, if accepted.<br><br>**Important:** Stating 'data available on request from the author' is not sufficient. If your data are only available upon request, select 'No' for the first question and explain your exceptional situation in the text box.<br><br>Do the authors confirm that all data underlying the findings described in their manuscript are fully available without restriction?	
**Describe where the data may be found in full sentences. If you are copying our sample text, replace any instances of XXX with the appropriate details.**<br><br>• If the data are **held or will be held in a public repository**, include URLs, accession numbers or DOIs. If this information will only be available after acceptance, indicate this by ticking the box below. For example: *All XXX files are available from the XXX database (accession number(s) XXX, XXX.*).<br>• If the data are all contained **within the manuscript and/or Supporting Information files**, enter the following: *All relevant data are within the manuscript and its Supporting Information files.*<br>• If neither of these applies but you are able to provide **details of access elsewhere**, with or without limitations, please do so. For example:<br><br>*Data cannot be shared publicly because of [XXX]. Data are available from the XXX Institutional Data Access / Ethics Committee (contact via XXX) for researchers who meet the criteria for access to confidential data.*<br><br>*The data underlying the results presented in the study are available from (include the name of the third party*	All relevant data are within the manuscript and its Supporting Information files.

and contact information or URL).<br>• This text is appropriate if the data are owned by a third party and authors do not have permission to share the data.<br><br>* typeset	
Additional data availability information:	

Manuscript Click here to access/download;Manuscript;Manuscript.docx

A NEW ARCHOSAUROMORPH FROM SOUTH AMERICA PROVIDES INSIGHTS ON THE EARLY DIVERSIFICATION OF TANYSTROPHEIDS

Tiane M. De-Oliveira[1*], Felipe L. Pinheiro[2], Átila Augusto Stock Da-Rosa[3], Sérgio Dias-Da-Silva[3], Leonardo Kerber[4]

[1] Programa de Pós-Graduação em Biodiversidade Animal, Universidade Federal de Santa Maria (UFSM), Prédio 13, Av. Roraima, 1000, 97105-900, Bairro Camobi, Santa Maria, Rio Grande do Sul, Brazil.

[2] Laboratório de Paleobiologia, Universidade Federal do Pampa, Av. Antônio Trilha 1847, 97300-162, São Gabriel, Brazil.

[3] Centro de Ciências Naturais e Exatas, Universidade Federal de Santa Maria (UFSM), Prédio 13, Av. Roraima, 1000,97105-900, Bairro Camobi, Santa Maria, Rio Grande do Sul.

[4]Centro de Apoio à Pesquisa Paleontológica da Quarta Colônia (CAPPA), Universidade Federal de Santa Maria (UFSM), Rua Maximiliano Vizzotto, 598, 97230-000, São João do Polêsine, Rio Grande do Sul, Brazil.

*Corresponding author

E-mail: tiane.m.deoliveira@gmail.com (TMO)

ABSTRACT

After the Permo-Triassic mass extinction, the archosauromorph fossil record is comparatively abundant and ecologically diverse. Among early archosauromorphs, tanystropheids gained considerable attention due to the presence of extreme skeletal adaptations in response to sometimes overspecialized lifestyles. The origin and early radiation of Tanystropheidae,

however, remains elusive. Here, a new Early Triassic archosauromorph is described and phylogenetically recovered as the sister-taxon of Tanystropheidae. The new specimen, considered a new genus and species, comprises a complete posterior limb articulated with pelvic elements~~ and an almost complete pes~~. It was recovered from the Sanga do Cabral Formation (Sanga do Cabral Supersequence, Lower Triassic of Paraná Basin, Southern Brazil), which have already yielded a typical Early Triassic vertebrate assemblage of temnospondyls, procolophonoids and scarce archosauromorph remains. This new taxon provides insights on the early diversification of tanystropheids, as well as being further evidence for a premature wide geographical distribution of this clade. The morphology of the new specimen is consistent with a terrestrial lifestyle, suggesting that this condition ~~as~~ plesiomorphic for Tanystropheidae.

Keywords: Archosauromorpha, Tanystropheidae, Triassic, Sanga do Cabral Formation, Western Gondwana.

INTRODUCTION

Archosauromorpha comprises an exceptionally diverse clade of diapsids originated during the Permian, progressively increasing its diversity throughout Mesozoic and Cenozoic eras. The most critical adaptive radiation of this clade took place following the aftermath of the Permian-Triassic mass extinction, resulting in a wide spectrum of occupation regarding both habitat and ecological niches [1,2]. After the Permian-Triassic crisis, the archosauromorph fossil record is considerably abundant and morphologically diverse, including highly specialized herbivores (rhynchosaurs), large apex predators (erythrosuchids), aquatic predators (phytosaurs), armored crocodile-like forms (aetosaurs), and gracile dinosaur precursors [1-3].

One of the early archosauromorph clades that better illustrates the morphological disparity of the group is the Tanystropheidae, which comprises *Macrocnemus* Nopcsa 1930, *Tanystropheus* Wild, 1973, *Amotosaurus* Fraser and Rieppel, 2006, *Langobardisaurus* Renesto, 1994 and *Tanytrachelos* Olsen, 1979) [2,4-6]. Tanystropheidae is remarkable for including sometimes bizarre representatives with extreme morphologies [5]. Members of this clade are recognizable by their long necks, composed of eight (*Macrocnemus*) to thirteen (*Tanystropheus*) moderately to extremely elongated cervical vertebrae with very long and low neural spines [7,8]. Overall, ~~T~~anystropheid *bauplan* is regarded as evidence of semiaquatic or even complete aquatic lifestyles [9-12]. However, recent studies failed to support a fully aquatic habit for tanystropheids, demonstrating that *Macrocnemus* was presumably terrestrial, whereas the lifestyle of the enigmatic *Tanystropheus*, the largest and most bizarre of all tanystropheids, remains enigmatic [8,13]. Tanystropheid record and related forms mostly come~~s~~ from the Middle/Late Triassic of Asia, Europe and North America [5,14], and the clade is exceptionally rare in Lower Triassic rocks (see [2,5,7,15,16]). Although the fossil record of Tanystropheidae was, until recently, restricted to the Northern Hemisphere, De-Oliveira *et al.* [17] described isolated cervical vertebrae that share synapomorphies with this clade from the Induan/Olenekian Sanga do Cabral Formation, which belongs the Brazilian portion of the Sanga do Cabral Supersequence. Besides, a humeral fragment compatible with Tanystropheidae was reported from Upper Permian strata of the Rio do Rasto Formation, Southern Brazil [18].

Based upon its tetrapod content, the Sanga do Cabral Formation is regarded as Lower Triassic, being correlated to the Katberg Formation of the South African Karoo Basin (*Lystrosaurus* Assemblage Zone) [19,20]. Although recent collection efforts substantially increased the number of archosauromorph specimens recovered from the Sanga do Cabral Formation (e.g. [17, 21, 22]), its diversity ~~in South American Lower Triassic~~ is still poor when compared to coeval deposits from South Africa, which have yielded the rhynchosaur

Noteosuchus Broom, 1925, the well-known *Prolacerta* Parrington, 1935, and the archosauriform *Proterosuchus* Broom, 1903 [23]. Nevertheless, the Sanga do Cabral Formation is one of the oldest Triassic sedimentary unit yielding fossil vertebrates from South America and provides a unique opportunity to study the biotic recovery after the P/T boundary.

This contribution provides a description and phylogenetic analysis of a new archosauromorph species from the Sanga do Cabral Formation, which provides insights on the hidden western Gondwanan archosauromorph divesity after the Permo-Triassic global crisis, adding information on the early distribution and lifestyle of tanystropheid-like forms.

INSTITUTIONAL ABBREVIATIONS

AMNH, American Museum of Natural History, New York; BP, Evolutionary Studies Institute, University of the Witwatersrand, South Africa; CAPPA, Centro de Apoio à Pesquisa Paleontológica da Quarta Colônia, São João do Polêsine, Rio Grande do Sul, Brazil; FMNH, Field Museum of Natural History, Chicago, Ilinois; GR, Ruth Hall Museum of Paleontology, Ghost Ranch, Mexico; MCSN, Museo Civico di Storia Naturali Milano, Italy; MCSNB, Museo Civico di Scienze Naturali Enrico Caffi, Bergamo, Italy; MCZ, Museum of Comparative Zoology, Cambridge, USA; MSNM, Museo di Storia Naturale, Milano, Italy; NHMUK, Natural History Museum of the United Kingdom, London; NMQR, National Museum Bloemfontein, Bloemfontein, South Africa; PIMUZ, Paleontological Institut and Museum, Zürich, Switzerland; SMNS, Staaliches Museum für Naturkunde Stuttgart, Stuttgart, Germany; TMM, Texas Memorial Museum, Austin, Texas, USA; UFSM, Universidade Federal de Santa Maria, Santa Maria, Rio Grande do Sul, Brazil; UWBM, Burke Museum of Natural History and Culture, USA; UMCZ, ~~Universidad de Murcia, Spain~~

METHODOLOGY

Material

The material, under collection number UFSM 11471 consists of a complete posterior limb with an articulated femur, tibia, and fibula, and pes. The specimen also preserves portions of the pelvic girdle, sacral and caudal vertebrae.

Phylogenetic Analysis

The phylogenetic analysis was carried out in order to access the affinities of UFSM 11471 with respect to other early archosauromorphs. UFSM 11471 was scored in the dataset of Pritchard *et al.* [6], as it includes a larger number of tanystropheids as terminal taxa when compared to other data matrixes. The analysis protocol consisted of heuristic searches of 1000 replications using random addition sequences followed by the Tree Bisection Reconnection (TBR) algorithm, retaining ten trees by replication. The dataset of Pritchard *et al.* [6] does not include *Jesairosaurus lehmani* Jalil, 1997, as an operational unit. The phylogenetic position of this enigmatic archosauromorph, formerly regarded as a member of "Prolacertiformes", has not been tested in recent analyses until the work of Ezcurra [2]. This later recovered *J. lehmani* as the sister-taxon to the Tanystropheidae, even though it differs from tanystropheids in several important features. As at this point a first-hand assessment of *J. lehmani* was not possible, we chose not to include this taxon as an OTU in the present analysis. After estimation of decay indexes (Bremer Support) (also using TNT), a search for suboptimal trees with one extra step was conducted. This second search aimed to verify the percentage of recovery, in suboptimal trees, of specific nodes with low decay indexes.

SYSTEMATIC PALEONTOLOGY

DIAPSIDA OSBORN, 1903 (*SENSU* LAURIN 1991)

ARCHOSAUROMORPHA HUENE, 1946 (*SENSU* GAUTHIER *ET AL*. 1988)

ELESSAURUS GONDWANOCCIDENS GEN. ET SP. NOV.

Holotype

UFSM 11471 – A partially articulated hind limb associated with axial elements, composed of femur, tibia, fibula, pelvic girdle bones, sacral and caudal vertebrae, as well as an almost complete pes. The specimen is permanently housed at the paleontological collection of the Laboratório de Paleobiologia e Estratigrafia of the UFSM, Santa Maria, Rio Grande do Sul, Brazil.

Etymology

Genus named after *Elessar*, meaning 'elf-stone' in the fictional language Quenya, created by J. R. R. Tolkien. In Tolkien's Middle Earth universe, Elessar is the name chosen by king Aragorn II, who, by his turn, is also known as Strider or 'longshanks'. The comparatively long zeugopodium of UFSM 11471 makes it a long-shanked animal, justifying the name. Termination *-saurus* from Greek, meaning 'lizard'. Species name derived from the supercontinent Gondwana and the Latin adjective *occidens*, 'from west', in a reference to the locality from where the new species was recovered.

Diagnosis

Elessaurus gondwanoccidens differs from all other archosauromorphs based upon a unique combination of characters: second sacral vertebral rib elongated and distally bifurcated, with a robust articular surface; transverse processes of the caudal vertebrae inclined posterodorsally; strongly sigmoidal femur; tibia and fibula longer than femur; metatarsals increase in size from the first to the fourth toe; fifth metatarsal short, with a proximal hook-shaped end; presence of a calcaneal tuber.

LOCALITY AND HORIZON

The specimen was collected at the locality known as Bica São Tomé, Sanga do Cabral Formation (Sanga do Cabral Supersequence, Paraná Basin), municipality of São Francisco de Assis, Rio Grande do Sul, Southern Brazil (29°36′ 56″ S, 55°03′ 10″ W) [21] (Fig 1). *Elessaurus gondwanoccidens* was collected in one of the five outcrops comprising the Bica São Tomé (outcrop 5 of Da-Rosa *et al*. [21]). An Induan-Olenekian age (251-247 Ma) [24] is inferred for this formation based on the presence of the parareptile *Procolophon trigoniceps* Owen, 1876, and comparisons with the *Lystrosaurus* Assemblage Zone of South African Karoo Basin [20,21,23,25]. *Elessaurus gondwanoccidens* represents the most complete postcranial skeleton so far recovered from this unit, as Sanga do Cabral fossils are often fragmentary, with rare occurrences of associated elements.

Fig 1. Type locality of *Elessaurus gondwanoccidens* (UFSM 11471). A. Geographic map with the location of *Elessaurus gondwanoccidens*, (São Francisco de Assis, Brazil); **B.** Simplified stratigraphic profile of the outcrop, showing the level where *Elessaurus gondwanoccidens* was found. Map was modified from Zerfass et al. [26] and stratigraphic profile modified from Da-Rosa et al. [21] and Pinheiro et al. [22]; silhouette adapted from Rieppel [14], showing bones preserved of UFSM 11471 in dark gray color.

DESCRIPTION AND COMPARISON

Elessaurus gondwanoccidens holotype is composed of an almost complete hindlimb associated with pelvic girdle bones and partially articulated sacral and caudal vertebrae (Fig 2). Although some elements show signs of compression (e.g. femur, tibia), all bones are close to a natural position, excepting for a slight displacement of some tarsal elements and distal phalanges missing in most digits. As will be discussed, the specimen is morphologically compatible with basal archosauromorphs, especially with Tanystropheidae.

Fig 2. Lateral view of *Elessaurus gondwanoccidens* (UFSM 11471) from Sanga do Cabral Formation (Lower Triassic), Brazil. Photograph and explanatory drawing respectively. Abbreviations: **fe**, femur; **ti**, tibia; **fi**, fibula; **il**, ilium; **sv**, sacral vertebra; **cv**, caudal vertebrae.

Vertebrae

Specimen UFSM 11471 preserves a complete second sacral vertebra associated with the first and second caudal elements (Fig 3). The first sacral comprises scattered fragments articulated to sacral II, which is well preserved and articulated to the ilium. The pleurapophysis of the second sacral vertebra is bifurcated distally, with a posterior process ending in a pointed tip. The distal end of the pleurapophysis is expanded and presents a wide triangular surface (as observed in dorsal view) which contacts the ilium.

Fig. 3. Dorsal view of *Elessaurus gondwanoccidens* (UFSM 11471) from Sanga do Cabral Formation (Lower Triassic), Brazil. Photograph and explanatory drawing respectively. Abbreviations: **sv2**, second sacral vertebra; **cv**, caudal vertebrae 1-3.

The elongated and distally bifurcated pleurapophysis of the second sacral vertebra resembles the condition observed in tanystropheids such as *Macrocnemus* [12,27]. This condition, however, is also present in *Trilophosaurus buettneri* Case, 1928 and *Prolacerta*, as well as in some extant lizards [5,12,14,28]. *Augustaburiania vatagini* shows an alternative condition, where sacral vertebrae present small sacral ribs that deviate laterally in the middle of the centrum [7]. *Tanystropheus longobardicus*, the best-known tanystropheid, lacks distally bifurcated sacral ribs, whereas *Amotosaurus rotfeldensis* presents the bifurcation of the second sacral rib more anteroposteriorly expanded than *Elessaurus gondwanoccidens*, which presents the posterior process of the second sacral rib distally sharp, ending in a pointed tip, whereas in *Prolacerta* this process is terminally blunt [6].

The anteriormost caudal vertebra has the same anteroposterior length of the second sacral and shows prominent transverse processes, projecting distinctly laterally to the pleurapophysis of the sacral vertebrae and the dorsal part of the ilium. In dorsal view, the transverse processes are posterolaterally directed. Although the transverse processes of the second caudal vertebra are scattered, they are slightly longer than those from the first caudal vertebra.

The anterior caudal vertebrae also present distinct transverse processes in *Tanystropheus* and *Langobardisaurus* [9,29]. *Tanytrachelos* does not present a bifurcate second sacral rib and, together with *Jesairosaurus lehmani*, it presents the transverse processes of the caudal vertebrae slightly inclined posterolaterally.

Pelvic Girdle

The pelvic girdle is fragmented, with its bones only partially preserved and exposed, being relatively small when compared to the large hindlimbs. The ilium is discernible in dorsolateral view, and it is not clear if both pubis and ischium are preserved. The ilium is expanded into a dorsal lamina that articulates with the large pleurapophysis of the sacral vertebra. Anteriorly to this, the ilium presents a strongly projected process. The dorsal margin of the iliac lamina is predominantly straight, with a horizontal orientation. The supracetabular surface is thickened, and the lateral surface of the acetabulum is roughly circular. The posterior process of the ilium is strongly developed, extending posteriorly to the acetabulum.

The pelvic girdles of *Prolacerta* and tanystropheids are similar to that of *Elessaurus gondwanoccidens* regarding the presence of a posterodorsally directed triangular blade on the ilium. Although the iliac lamina of *Elessaurus* is partially broken, it is possible to discern a short preacetabular process similar to that observed in *Macrocnemus bassanii* (specimen T 2472). This is a robust process in this specimen, being also present (albeit less pronounced) in *Tanystropheus longobardicus* (specimen MSNM BES SC 1018). Conversely, in *Prolacerta* the anterior margin of the ilium is convex, the same occurring in *Jesairosaurus lehmani* [12,14,15,30,31].

Femur

With a total size of 64.85 mm, the femur is slightly shorter than the tibia and fibula. Its proximal end is poorly preserved and strongly compressed. The distal end of the femur is 16,55 mm in width, whereas the proximal part measures 15.44 mm. It is a gracile bone, being the ratio between the transversal width of the distal end and the total length of the bone 3,91. Despite it

shows signs of preservational compression, the femur is strongly sigmoid in lateral view, differing from the specimen GR-304 described by Pritchard *et al*. [5], which is nearly straight, turning distally from the surface of the proximal head.

Both *Tanystropheus longobardicus* (MSNM BES SC 265) and *Tanytrachelos* (YPM 7622) present a more subtle femur curvature when compared to *Elessaurus gondwanoccidens* [2,5,12,32,33], which resembles *Macrocnemus bassanii* (T 4355) and *Augustaburiania*, as both present gracile and strongly sigmoidal femora [7,14].

Probably due to poor preservation, the proximal surface of the femur has a quadrangular outline. The femoral head appears to be confluent with the shaft. The dorsolateral margin of the proximal portion of the femur is smooth and featureless, as is the transition between the femoral head and diaphysis. The femoral head is weakly expanded in tanystropheids (e.g., *Tanytrachelos ahynis*, AMNH FARB 7206, GR 301). In other basal archosauromorphs such as *Azendohsaurus madagaskarensis* Flynn *et al*. 2010 (UA 7-20-99-653), the proximal end is moderately expanded relative to the midshaft, as it is in some rhynchosaurs and early archosauriforms (e.g., *Proterosuchus alexanderi* Hoffman, 1965, NMQR 1484; *Erythrosuchus africanus* Gower, 1996 NHMUK 3592) [4]. The internal trochanter is present as a ridge-shaped process that defines a relatively wide intertrochanteric fossa, converging to the proximal end. The internal trochanter is continuous with the proximal articular surface. The transition from the plesiomorphic condition of a proximal trochanter including an inner trochanter and a posterior trochanter (e.g. *Erythrosuchus africanus*; *Trilophosaurus buettneri*) to a large fourth trochanter and a larger trochanter (e.g. *Alligator*, Hutchinson, 2001; dinosaurs), occurs within archosauriforms [1,5,33]. Thus, the presence of an internal trochanter allied to the absence of a fourth trochanter strongly supports nesting of *Elessaurus gondwanoccidens* in a clade outside Archosauriformes. A well-developed internal trochanter projecting from the proximal end of the femur is present in early archosauromorphs, including tanystropheids (e.g. *Macrocnemus*

bassanii and *Tanystropheus longobardicus*). An internal trochanter that does not reach the proximal surface of the femur is evident in rhynchosaurs and some archosauriforms, such as *Proterosuchus fergusi* Broom, 1903 and *Erythrosuchus africanus* [2,5,34,35].

Despite its compression, *Elessaurus gondwanoccidens*´s femur is slightly widened distally. Distally expanded femora (although in a lesser degree) occur in *Prolacerta broomi* (BP/1/2676) and tanystropheids (e.g. *Tanystropheus*, Wild [9]; GR 301, Pritchard *et al*. [5]).

The distal end of the femur is marked by two delineated, unequal distal condyles, with the lateral larger than the medial one. They are distinctly expanded beyond the circumference of the femoral shaft. In distal view, the fibular condyle has a subtriangular lateral surface.

Tibia and Fibula

The poor preservation of the tibia hinders a proper morphological assessment of this bone. Tibia and fibula present both a length about 12% greater than the femur. The length relationship between tibia and fibula is considered an important phylogenetic feature in basal archosaurs (see Ezcurra [2]). Among those, the tibia is longer than the femur in basal pterosaurs (e.g. *Preondactylus* Wild, 1984), *Lagerpeton* Romer, 1971, *Dromomeron* Irmis *et al*. 2007, *Marasuchus* Sereno and Arcucci, 1994, *Pseudolagosuchus* Arcucci, 1987, basal ornithischians, *Eoraptor* Sereno *et al*. 1993 and most basal dinosauromorphs. [1]. Within non-archosauriforms, this characteristic is prominent among some tanystropheids (e.g. *Macrocnemus*). Compared to the forelimbs, the hindlimbs of *Macrocnemus* are strongly elongated [14], which is mainly acquired by an elongation of the tibia/fibula.

Besides, a short femur relative to the tibia and fibula is also present in *Prolacerta* (AMNH 9502, BP / 1/2676) [30]. Although also having a proportionally short femur, *Prolacerta* specimen described by Spiekman [31] (UWBM 95529) shows a proportionally longer femur

when compared to *Macrocnemus* and *Elessaurus*. UWBM 95529 has a femur measuring 72.4 mm, whereas the tibia is 69.3 mm in length.

Tibia and fibula at least 20% longer than the femur is one of the synapomorphies diagnosing *Macrocnemus* [5]. There are reports of proportional differences [36,37], among *Macrocnemus bassanii*, *Macrocnemus fuyuanensis*, and *Macrocnemus obristi*. Some authors even suggest that these differences may be related to sexual dimorphism [13]. The tibia of *Elessaurus gondwanoccidens* is much thicker than the fibula, and has its distal end articulated with mesopodial elements. The distal end of the tibia is fragmented, what may be a result of pre-burial fracturing. Although the tibia is severely damaged, it is possible to observe a small groove in the lateral surface of its distal end, a feature only observed in some dinosaurs and proterochampsids (e.g. *Chanaresuchus* Reig, 1971, *Tropidosuchus* Arcucci, 1990) [1]. In the context of non-archosauriforms, thus, this may potentially be an autapomorphy of the new taxon described herein. However, this character might be also an artifact of poor preservation. The proximal end of the fibula is fragmented and compressed in proximal view, being rounded and symmetrical in lateral view. The area for insertion of the *M. iliofibularis* is evident by the presence of a distinct but low tubercle located near the proximal portion of the bone. The distal portion of the fibula is slightly asymmetrical in lateral view.

Pes

Metatarsals and phalanges are articulated and arranged close to natural position, with only a slight displacement of some elements. *Elessaurus gondwanoccidens* preserves the metatarsals I-V, but phalanges of the fifth digit are missing. The metatarsals increase in length from the first to the fourth digit, where the metatarsal IV is distinctly larger than the III (Fig 4). The fourth digit of non-archosauriform archosauromorph pes (e.g., rhynchosaurs, *Trilophosaurus*, *Prolacerta*) is the longest, whereas digit 3 is the longest in *Euparkeria* Broom, 1913 (UMCZ

T692) and all archosaurs in which this character can be accessed [1,2,38]. Metatarsals increase in size from the first to the fourth toe in *Macrocnemus bassanii* (T 2477; A III/208; T 2472), *Amotosaurus* (SMNS 54810), *Prolacerta*, and *Langobardisaurus* [14,16,29,39]. The metatarsus of *Tanystropheus* is asymmetrical, although not in the same degree as *Macrocnemus* and *Langobardisaurus* as, in *Tanystropheus*, the third metatarsal is the longest. *Tanytrachelos* (YPM 7540) apparently has a similar metatarsal configuration [12,32] as *Tanystropheus*.

Fig 4. Plantar (A) and posteroplantar (B) views of the pes of *Elessaurus gondwanoccidens* (UFSM 11471) from Sanga do Cabral Formation (Lower Triassic), Brazil. Photographs and explanatory drawings respectively. Abbreviations (**A**): **ca**, calcaneum; **dt 4**, distal tarsal 4; **mt**. metatarsal 1-5; **d3 - d4**, digits; **ph**, phalange. (**B**) **ti**, tibia; **fi**, fíbula; **ca**, calcaneum; **as**. astragalus; **tu**, calcaneal tuber; **mt**, metatarsal 5; **dt 1**, distal tarsal 1; **ce**, centrale; **dt 3**, distal tarsal 3; **dt 4**, distal tarsal 4.

The fifth metatarsal is short and has a proximal hook-shaped end: its proximal process is abruptly flexed and, as a result, the metatarsal is “L”-shaped in ventral view. This morphology is observed in *Macrocnemus bassanii*, allokotosaurs (e.g. *Pamelaria dolichotrachela* Sen, 2003, *Azendohsaurus madagaskarensis*), a few basal rhynchosaurs (e.g. *Noteosuchus colletti* Watson, 1912), *Boreopricea funerea* Tatarinov, 1978, *Prolacerta broomi*, and some archosauriforms (e.g. *Proterosuchus fergusi* Broom, 1903) [1,2]. Among tanystropheids, the morphology of metatarsal V from *Elessaurus gondwanoccidens* resembles the condition displayed by *Macrocnemus* (PIMUZ T AIII / 208) [14] and differs from that of *Langobardisaurus* and *Tanystropheus* (MSNM V 3730) [12], as these present a less pronounced hook-shaped element. The metatarsals diverge from the tarsus distally, but overlap proximally.

In digits I and II the distal phalanges are not preserved and the digits III and IV present two middle and one distal phalange. The lack of some distal phalanges prevents an exact account of the phalangeal formula.

Six tarsals are preserved in *Elessaurus gondwanoccidens*, including the proximal elements (astragalus and calcaneum), and four ossifications identified here as the distal tarsal elements I, III, IV, and the centrale (Fig 4B). Excepting the centrale, these elements are displaced laterally towards the calcaneum, distal to the tibia and proximal to the metatarsals I and II. A fifth distal tarsal is missing. From distal elements, fourth and centrale are the largest. Distal tarsal IV is located between the astragalus and calcaneus, proximal to metatarsals III and IV, whereas distal tarsals III and I are placed medial to the centrale. Four distal tarsals occur in most early archosauromorphs (e.g., *Mesosuchus browni* Watson, 1912, SAM 7416; *Protorosaurus speneri* Meyer, 1832; *Trilophosaurus buettneri*, TMM 31025- 140) [4]. *Macrocnemus bassanii* presents four distal tarsals, one being the centrale. However, only three distal tarsals occur in *Macrocnemus fuyuanensis* and *Amotosaurus*, and only two in *Tanystropheus longobardicus* (MCSN BES SC 1018; MCSN V 3730) [4,14,40,41]. *Prolacerta broomi* [BP/1 2676] [30] was described as having a centrale in close contact with the mesial surface of the astragalus, besides four distal elements, of which the first three are small and fragmented. Colbert [39] argued that the centrale is absent in AMNH 9502, in contrast to Gow's [30] description for *Prolacerta*. However, according to Colbert [39], this bone had likely been lost during fossilization. *Prolacerta* specimen UWBM 95529 [31] preserves a central bone, in agreement with initially stated by Gow [30] being, therefore, similar to what is observed in *Elessaurus gondwanoccidens*. No centrale bones appear to be present in *Tanystropheus longobardicus* (MCSN V 3730), although the distal tibial articular surface is wide. In *Tanystropheus*, the astragalar body and centrale thus are possibly indistinguishably fused [4]. The presence of a cartilaginous centrale in *Tanystropheus* also remains highly conjectural [12].

Langobardisaurus and *Macrocnemus* have the area distal and/or medial to the astragalus occupied by an ossified centrale [12]. According to Rieppel [14], in *Macrocnemus*, the tibia articulates with the astragalus, bearing a distinct articular facet on its medial side. This facet forms the proximal part of an embayment completed by the centrale and distal tarsal I, within which accommodates the tibia during the stride phase when maximal propulsive force is applied.

In *Elessaurus gondwanoccidens*, astragalus and calcaneum are unfused and lack a perforating foramen. This contrasts with *Prolacerta* [31], in which it is possible to observe a perforating foramen between these elements (UWBM 95529). A perforating foramen is absent in *Langobardisaurus* (MCSNB 2883, MCSNB 4870, MFSN 1921, MFSN 26829) and *Tanytrachelos* (VMNH 120015, YPM 8600), although this may be consequence of small size, or even an artifact of preservation [5,42]. According to Rieppel *et al.* [43] this foramen is absent in *Tanystropheus cf. Ta. longobardicus*. A larger perforating foramen is present in *M. bassanii* and *Amotosaurus* [5,14,16]. The surface for tibial articulation of the calcaneum is slightly rounded, and the articular surface of the distal tarsal IV is concave. The articular surface for the astragalus appears to be continuous with the articulation of the distal tarsals.

The calcaneum is quadrangular in lateral view, being wider on its anteroposterior axis than proximo-distally, becoming “L”-shaped distal to the fibula. The proximal surface of the calcaneum is marked by the presence of a rough tuberosity, the calcaneal tuber. In ~~the~~ proximal view, the calcaneal tuber is “square”-shaped, longer proximo-distally than dorsoventrally, and the distal part presents a curvature. The tuber is proximo-distally longer than dorsoventrally tall, in similar proportions to ~~the~~ observed in most early archosauromorphs (e.g., *Tanytrachelos ahynis*, GR 306; *Trilophosaurus buettneri*, TMM 31025-140; *Azendohsaurus*, FMNH PR 2776) [4]. There is a notch between the main body of the calcaneu~~s~~ and the tuber. Among tanystropheids, the calcaneal tuber is only present in *Tanytrachelos ahynis* Olsen, 1979,

although it is a typical characteristic of several clades within Archosauriformes. According to Nesbitt *et al*. [4], a fibular facet continuous with the lateral tuber is present in some *Trilophosaurus* (AMNH FARB 30836), *Proterosuchus alexanderi* Hoffman, 1965 (MCZ 4301), and *Erythrosuchus africanus* (NHMUK R3592). Pritchard *et al*. [5] assigned specimen GR 306 to *Tanytrachelos* based on features of the calcaneus, such as the strongly laterally expanded distal end and the distal curvature of the calcaneal tuber. The calcaneal tuber of GR 306 is still larger than observed in other taxa. This structure is well evident in *Elessaurus gondwanoccidens* and compatible with that observed in GR 306, as the proximal surface of the calcaneum is marked by the development of its lateral margin, characterizing a rough tuberosity. Nesbitt *et al*. [4] noted a similar condition to *Tanytrachelos ahynis* (GR 306) in *Azendohsaurus* (FMNH PR 2776).

PHYLOGENETIC ANALYSIS

Our analysis recovered only one MPT with 1120 steps (consistency index 0.321 and retention index 0.642), in which *Elessaurus gondwanoccidens* is the sister taxon of Tanystropheidae (Fig 5), this later being a node-based clade comprising the most recent common ancestor of *Macrocnemus, Tanystropheus,* and *Langobardisaurus* and all its descendants [44]. *Elessaurus gondwanoccidens* shares some similar character states with Tanystropheidae, as the pleurapophysis of the second sacral vertebra distally bifurcated (character-state 131:0→1), tibia and fibula with a total length slightly larger than the femur (character-state 516:0→2, [2]) and "hook"-shaped fifth metatarsal (character-state 197:0→1). The node (*Elessaurus gondwanoccidens* + Tanystropheidae) is supported by the presence of a distally sharp posterior process of the second sacral rib and the transverse process of the anterior caudal vertebrae angled posterolaterally. In addition, the new specimen presents some features only found in more specialized representatives within Tanystropheidae, such as the presence of

a well-developed calcaneal tuber with a rough lateral margin. Albeit Bremer support for *Elessaurus gondwanoccidens* plus Tanystropheidae is low (=1), this particular node was present in 66% of the 36 sub-optimal trees with one extra step recovered by the second analysis.

Fig 5. A- Archosauromorph phylogeny showing the recovered position of *Elessaurus gondwanoccidens* (UFSM 11471), from the matrix of Pritchard *et al*. [6] and the geographic distribution maps for Tanystropheidae through time (green circles) and the brazilian fossil record (red star) (data from the Paleobiology Database, https://paleobiodb.org/#/) (B, early Triassic; C, middle Triassic; D, late Triassic).

DISCUSSION

Taxonomic remarks

The specimen herein described is morphologically compatible with non-archosauriform archosauromorphs, in a close relationship with Tanystropheidae supported by several characters (see above).

Recent phylogenetic analyzes recovered tanystropheids and *Jesairosaurus lehmani* more closely related to each other than to other archosauromorphs [2]. Albeit the matrix of Pritchard *et al*. [6], used in this work, does not include *Jesairosaurus lehmani* (see above), *Elessaurus gondwanoccidens* differs from *J. lehmani* in several characters. According to Ezcurra [2], it is not possible to observe the presence of an internal trochanter or fourth trochanter in *J. lehmani*. Besides, the calcaneum of *J. lehmani*, although poorly preserved, lacks a calcaneal tuber (present in *Elessaurus gondwanoccidens*), and the distal end of the femur does not taper distally in dorsal view. Although the phylogenetic relationship of *J. lehmani* with

Elessaurus gondwanoccidens still need to be tested, the presence of the mentioned characters in the later may indicate that *Elessaurus gondwanoccidens* is more closely related to Tanystropheidae than to *J. lehmani*.

Among basal archosauromorphs, tibia/fibula longer than the femur is observed in Tanystropheidae and *Prolacerta*. Although *Prolacerta* is one of the best represented early archosauromorphs, its postcranial morphology remains poorly known, and most studies have focused on cranial anatomy. Its postcranial anatomy, however, was discussed by Gow [30], Colbert [39] and, more recently, Spiekman [31]. Despite the overall similarity among *Elessaurus gondwanoccidens*, Tanystropheidae (e.g. *Macrocnemus*), and *Prolacerta*, the specimen herein described is more consistent with Tanystropheidae than to *Prolacerta*. A foramen perforating the astragalus/calcaneum is present in *Prolacerta* [31], whereas *Elessaurus gondwanoccidens* does not exhibit this feature. The good preservation of the proximal tarsals in *Elessaurus gondwanoccidens* suggests that the absence of this foramen is not a preservation bias. Moreover, the posterior process of the bifurcated second sacral rib of *Elessaurus gondwanoccidens* is distally sharp, whereas in *Prolacerta* this process is blunt [6].

Teyujagua paradoxa Pinheiro *et al*. 2016 lacks comparable elements with *Elessaurus gondwanoccidens*, this species was recovered as the sister taxon to the Archosauriforms and is thus more closely related to proterosuchids than *Prolacerta* and, consequently, *Elessaurus gondwanoccidens* [22].

Biogeographical Implications

In the aftermath of the Permian–Triassic crisis, earliest Triassic continental communities were extremely impoverished, including few small and unspecialized tetrapod taxa [7,23,45]. Adaptive radiation of early archosauromorphs, including tanystropheids, possibly occurred

already during the Early Triassic. Although the most abundant and the better-known records of this group belong to the Middle Triassic, (Ladinian) of Switzerland and Italy, this group also have rare records in Lower Triassic strata. *Amotosaurus rotfeldensis* and *Augustaburiania vatagini* are thus far the earliest nominal taxa of Tanystropheidae, being known from non-marine rocks from Germany and Russia [5,7,16].

Among the genera abovementioned, only *Macrocnemus* and *Tanystropheus* are known to occur in both the western and eastern Tethyan province, with specimens of *M. fuyuanensis* and *T. longobardicus* from Europe being slightly older (Late Anisian to early Ladinian) than Chinese ones (Ladinian) [13]. Jaquier *et al.* [13] and Pritchard *et al.* [5] proposed that during the Late Early Triassic, tanystropheid reptiles first evolved from their Late Permian and Early Triassic ancestors in central Pangea and dispersed afterwards along the western and eastern margins of the Tethys Ocean during the Middle Triassic.

Elessaurus gondwanoccidens, together with the specimens reported by De-Oliveira *et al.* [17] comprises the known record of Tanystropheidae-like archosauromorphs in South America. The recovery of *Elessaurus gondwanoccidens* as the sister taxon of Tanystropheidae suggests the diversification of tanystropheid-related animals in South America still during the Early Triassic. Furthermore, it corroborates an early diversification of the group in central Pangea, possibly with a Gondwanan origin, reaching cosmopolitan distribution already during the early Mesozoic.

The transition of tanystropheids from terrestrial to semiaquatic and, then, aquatic and even marine habitats throughout Triassic was probably connected with the diversification of the terrestrial biota, niche packing, increasing competitive pressure within continental communities, and diversification of predators [7]. *Elessaurus gondwanoccidens* may represent one of the oldest Tanystropheidae-related archosauromorphs to this date. The fact that the new specimen was collected in a depositional environment of ephemeral fluvial systems in an arid

landscape, quite distinct from the marine deposits where tanystropheids are usually found, further corroborates the ecological plasticity of the clade.

Tanystropheid ecology and the lifestyle of *Elessaurus gondwanoccidens*

Tanystropheidae is a clade mainly characterized by a long neck formed by elongate cervical vertebrae with low neural spines [5,7,12,36]. Some tanystropheids possibly had terrestrial or semi-aquatic habits, whereas more specialized forms may have been fully aquatic. Tanystropheid lifestyle, however, is still a matter of controversy. *Macrocnemus* and *Langobardisaurus* were supposedly terrestrial [8,14,46]. The association of *Tanytrachelos ahynis* with numerous fossil fishes, a lacustrine insect assemblage, abundant branchiopods, and phyllocarids, suggest~~s~~ it was aquatic, living in freshwater environments [32]. Some researchers indicate a digitigrade stance in the pes of *Tanytrachelos* [47], *Macrocnemus* [48] and *Langobardisaurus* [46], and ~~to~~ a possibly bipedal posture~~,~~ during rapid locomotion – as previously stated by Rieppel [14] for *Macrocnemus* – or even while standing and walking [12]. The foot of *Macrocnemus* appears to suit terrestrial locomotion, a conclusion further supported by the structure of its pelvic girdle [14]. The lifestyle of *Tanystropheus*, the largest and most bizarre of all tanystropheids, is still debatable. Recent osteological analyses do not support a fully aquatic habit for this animal [49], even though many skeletal features indicative of terrestrial habits in *Macrocnemus* are absent in *Tanystropheus*: as it does not present bifurcating pleurapophyses on the second sacral vertebra; the preacetabular process is absent in the ilium; tarsal ossifications show a greater degree of reduction; the hooked fifth metatarsal is less distinctly differentiated; finally, the metatarsus is far less asymmetrical [14]. One of the most striking features would be the much longer neck present in *Tanystropheus*. Renesto [11] emphasized that the neck of *Tanystropheus* was rather mobile and held horizontally or considerably raised. Tschanz [10], drawing a comparison with extant reptiles (*Iguana* and

Varanus), concluded that the neck of *Tanystropheus* would be almost inflexible, indicating a fully aquatic habit. Moreover, the reduced size of the forelimb suggests that it had not a major contribution to any kind of locomotion [11,12]. Reassessing this genus, Nosotti [12] regarded *Tanystropheus* as an aquatic animal with close terrestrial ancestors, living in shallow waters and probably returning to land for reproduction. Recently, some authors have proposed that the locomotion of *Tanystropheus* is consistent both with feeding on aquatic prey and with a semi-aquatic life-style in the near-shore environments [8]. The skeletal anatomy of *Tanystropheus* is unique, and there is no analogs in present-day or extinct animals. This peculiar structure, together with the impressive overall size of *Tanystropheus* (the largest individuals of *T. longobardicus* reached up to five meters in length) renders this animal a weird appearance, being still a palaeoecological and functional enigma [8].

At least one specimen of *Tanytrachelos* (AMNH FARB 7206) [5] bears a calcaneal tuber similar to the one of *Elessaurus gondwanoccidens*. Some authors have argued the absence of calcaneal tuber in tanystropheids (e.g., [12,14]. The absence of this tuber in more specialized forms might be attributable to an aquatic lifestyle [5,14]. Based on the presence of a calcaneal tuber, the hooked fifth metatarsal and the distally bifurcating pleurapophyses on the second sacral vertebra, we propose a terrestrial habit for *Elessaurus gondwanoccidens*, similar to what is argued for *Macrocnemus* and *Tanytrachelos*, and distinct from what usually proposed for *Tanystropheus*. This interpretation agrees with some authors [5,7,12] in which tanystropheids or close relatives were able to inhabit a wide range of climatic conditions and that, although possessing most of its representatives with affinities to an aquatic lifestyle, this group possibly presents close ancestors with terrestrial habit.

The depositional model of the locality from where *Elessaurus gondwanoccidens* was collected also supports interpretation based on its morphology. The Sanga do Cabral Formation is characterized as a system of ephemeral, high energy river channels with wide and extensive

alluvial plains, containing a rich assemblage of terrestrial and aquatic tetrapods composed of temnospondyls, procolophonoids, and archosauromorphs [20,21,50]. Considering both morphology and the environment described for the locality of Bica São Tomé, this animal would probably be terrestrial, inhabiting the vicinities of shallow waters and low-sinuosity river environments.

CONCLUSIONS

Until recently, the Sanga do Cabral Formation provided only few remains assigned to Archosauromorpha indet. Now, at least two independent lineages were reported for this unit ([17,21,22] and this work). Although rare, these fossils demonstrate that archosauromorphs were already diversified in the Early Triassic of western Gondwana. *Elessaurus gondwanoccidens* is here recovered as the sister taxon of Tanystropheidae and was collected from rocks reminiscent of continental environments dominated by ephemerous water bodies. Most representatives of Tanystropheidae (e.g. *Tanystropheus*) belong to marine environments. The results of the present work suggest that terrestrial mode of life was plesiomorphic for Tanystropheidae and maintained by some of its representatives (e.g. *Macrocnemus*). The record of Tanystropheidae-related taxa in Permian and Lower Triassic layers from South America indicates a premature wide distribution of this clade, with a possible Gondwanan origin.

ACKNOWLEDGEMENTS

This study was financed by the Coordenação de Aperfeiçoamento de Pessoal de Nível Superior – Brasil (CAPES) – Finance Code 001. Fundação de Amparo à Pesquisa do Estado do Rio Grande do Sul (FAPERGS 17/2551-0000816-2 to LK) and Conselho Nacional de Desenvolvimento Científico e Tecnológico (CNPq 422568/2018-0 to LK). SDS is supported

by CNPq (research grant, process number 306352/2016-8). FLP is supported by CNPq (process numbers 407969/2016-0, 305758/2017-9) and FAPERGS (process number 16/2551-0000271-1). Rodrigo Müller and Flavio Pretto (Centro de Apoio à Pesquisa Paleontológica da Quarta Colônia, Rio Grande do Sul, Brazil) made useful comments on description of the specimen and Lívia Roese Miron for making the drawnings.

REFERENCES

1. Nesbitt SJ. The Early Evolution of Archosaurs: Relationships and the Origin of Major Clades. Bulletin of the American Museum of Natural History. 2011; 352:1-292.

2. Ezcurra MD. The phylogenetic relationships of basal Archosauromorphs, with an emphasis on the systematic of Proterosuchian Archosauriforms. PeerJ. 2016; 4:e1778

3. Ezcurra MD, Butler RJ. The rise of the ruling reptiles and ecosystem recovery from the Permo-Triassic mass extinction. Proc. R. Soc. B. 2018; 285:20180361.

4. Nesbitt SJ, Flynn JJ, Pritchard AC, Parrish JME, Ranivoharimanana L, Wyss AR. Postcranial anatomy and relationships of *Azendohsaurus madagaskarensis*. Bulletin of the American Museum of Natural History. 2015; 398:1-126.

5. Pritchard AC, Turner AH, Nesbitt SJ, Irmis RB, Smith ND. Late Triassic tanystropheids (Reptilia, Archosauromorpha) from northern New Mexico (Petrified Forest Member, Chinle Formation) and the biogeography, functional morphology, and evolution of Tanystropheidae. Journal of Vertebrate Paleontology. 2015; 35:e911186.

6. Pritchard AC, Gauthier JA, Hanson M, Bever GS, Bhullar Bhart-AnjanS. A tiny Triassic saurian from Connecticut and the early evolution of the diapsid feeding apparatus. Nature Communications. 2018; 9:1213.

7. Sennikov AG. New Tanystropheids (Reptilia: Archosauromorpha) from the Triassic of Europe. Paleontological Journal. 2011; 45:90-104.

8. Renesto S, Saller F. Evidences for a semi aquatic life style in the Triassic diapsid reptile Tanystropheus. Riv. It. Paleontol. Strat. 2018; 124:23-34.

9. Wild R. Die Triasfauna dr Tessiner Kalkalpen. XXIII Tanystropheus longobardicus (Bassani) (Neue Ergebnisse). Abhandlungen der Schweizerischen Paläontologischen Gesellschaft. 1973; 95:1-162.

10.Tschanz K. *Tanystropheus*- An unusual reptilian construction. In: Konstruktionsprinzipien lebender und ausgestorbener Reptilien. Konzepte. Stuttgart. 1985; 4:169-178.

11. Renesto S. A new specimen Tanystropheus (Reptilia Protorosauria) from the Middle Triassic of Switzerland and the ecology of the genus. Riv. It. Paleont. Strat. 2005; 111:377-394.

12. Nosotti S. *Tanystropheus longobardicus* (Reptilia, Protorosauria): Re-interpretations of the anatomy based on new specimens from the Middle Triassic of Besano (Lombardy, Northern Italy). Memorie Della Societa Italiana di Scienze Naturali e del Museo Civico di Storia Naturale di Milano. 2007; 35.

13. Jaquier VP, Fraser NC, Furrer H, Scheyer TM. Osteology of a New Specimen of *Macrocnemus aff. M. fuyuanensis* (Archosauromorpha, Protorosauria) from the Middle Triassic of Europe: Potential Implications for Species Recognition and Paleogeography of Tanystropheid Protorosaurs. Front. Earth Sci. 2017; 5:91.

14. Rieppel O. The hind limb of *Macrocnemus bassani* (Nopcsa) (Reptilia, Diapsida): development and functional Anatomy. Journal of Vertebrate Paleontology. 1989; 9:373-387.

15. Jalil N-E. A new prolacertiform diapsid from the Triassic of North Africa and the interrelationships of the Prolacertiformes. Journal of Vertebrate Paleontology. 1997; 17:506-525.

16. Fraser NC, Rieppel O. A new protorosaur (Diapsida) from the Upper Buntsandstein of the Black Forest, Germany. Journal of Vertebrate Paleontology. 2006; 26:866-871.

17. De-Oliveira TM, Oliveira D, Schultz CL, Kerber L, Pinheiro FL. Tanystropheid archosauromorphs in the Lower Triassic of Gondwana. Acta Palaeontologica Polonica. 2018; 63:713–723.

18. Martinelli AG, Francischini H, Dentzien-Dias PC, Soares MB, Schultz CL. The oldest archosauromorph from South America: postcranial remains from the Guadalupian (mid-Permian) Rio do Rasto Formation (Paraná Basin), southern Brazil. Historical Biology. 2016; 29:76–84.

19. Cisneros JC. Taxonomic status of the reptile genus Procolophon from the Gondwanan Triassic Palaeontol. Afr. 2008; 43:7-17.

20. Dias-Da-Silva, S, Pinheiro FL, Da-Rosa AAS, Martinelli AG, Schultz CL, Silva-Neves E, Modesto SP. Biostratigraphic reappraisal of the Lower Triassic Sanga do Cabral Supersequence from South America, with a description of new material attributable to the parareptile genus *Procolophon*. Journal of South American Earth Sciences, 2017; 79:281-296.

21. Da-Rosa AAS, Piñeiro G, Dias-Da-Silva S, Cisneros JC, Feltrin FF, Neto LW. Bica São Tomé, um novo sítio fossilífero para o Triássico Inferior do Sul do Brasil. Revista Brasileira Paleontologia. 2009; 12:67-76.

22. Pinheiro FL, França MAG, Lacerda MB, Butler RJ, Schultz CL. An exceptional fossil skull from South America and the origins of the archosauriform radiation. Scientific Reports. 2016; 6:22817.

23. Botha JA, Smith RMH. Rapid vertebrate recuperation in the Karoo Basin of South Africa following the End-Permian extinction, Journal of African Earth Sciences. 2006; 45:502–514.

24. Cohen KM, Finney SC, Gibbard PL, Fan J-X. The ICS International Chronostratigraphic Chart. Episodes. 2013; 36:199-204.

25. Dias-Da-Silva S, Modesto SP, Schultz CL. New material of *Procolophon* (Parareptilia: Procolophonoidea) from the Lower Triassic of Brazil, with remarks on the ages of the Sanga do Cabral and Buena Vista formations of South America. Earth Sci. 2006; 43:685-1693.

26. Zerfass H, Lavina EL, Schultz CL, Garcia AJV, Faccini UF, Chemale FJr. Sequence stratigraphy of continental Triassic strata of Southernmost Brazil: a contribution to southwestern Gondwana paleogeography and paleoclimate Sedimentary Geology. 2003; 161:85–105.

27. Gregory JT. Osteology and relationships of *Trilophosaurus*. University of Texas Publication. 1945; 4401:273-359.

28. Hoffstetter R, Gasc J-P. Vertebrae and ribs of modern reptiles; pp. 209–301 In: C. Gans (ed.), Biology of the Reptilia, Morphology. Academic Press, London and New York. 1969.

29. Saller F, Renesto S, Dalla Vecchia FM. First record of *Langobardisaurus* (Diapsida, Protorosauria) from the Norian (Late Triassic) of Austria, and a revision of the genus. Neues Jahrbuch für Geologie und Paläontologie – Abhandlungen. 2013; 268:83-95.

30. Gow CE. The morphology and relationships of *Youngina capensis* Broom and *Prolacerta broomi* Parrington. Palaeontologia Africana. 1975; 18:89-131.

31. Spiekman SNF. A new specimen of *Prolacerta broomi* from the lower Fremouw Formation (Early Triassic) of Antarctica, its biogeographical implications and a taxonomic revision. Scientific Reports. 2018; 8:17996.

32. Olsen PE. A new aquatic Eosuchian from the Newark Supergroup (Late Triassic-Early Jurassic) of North Carolina and Virginia. Pastil/a New Haven. 1979; 176:1-14.

33. Nesbitt SJ, Stocker MR, Small BJ, Downs A. The osteology and relationships *of Vancleavea campi* (Reptilia: Archosauriformes). Zoological Journal of the Linnean Society. 2009; 157:814-864.

34. Cruickshank A. The proterosuchian thecodonts. In: Joysey, K. A., Kemp, T.S., eds. Studies in vertebrate evolution. Edinburgh: Oliver and Boyd. 1972; 89-119.

35. Gower DJ. Osteology of the early archosaurian reptile *Erythrosuchus africanus* Broom. Annals of the South African Museum. 2003; 110:1-84.

36. Li C, Zhao L-J, Wang L-T. A new species of *Macrocnemus* (Reptilia: Protorosauria). Linnean Society. 2007; 174:571-588.

37. Fraser N, Furrer H. A new species of *Macrocnemus* from the Middle Triassic of the eastern Swiss Alps. Swiss J Geosci. 2013; 106:199–206.

38. Gauthier JA. A cladistic analysis of the higher categories of the Diapsida. PhD thesis (University of California) Berkeley. 1984; 564.

39. Colbert EH. The Triassic reptile *Prolacerta* in Antarctica. American Museum Novitates. 1987; 2882:1-19.

40. Gottmann-Quesada A, Sander PM. A redescription of the early archosauromorph *Protorosaurus speneri* (Meyer, 1832) and its phylogenetic relationships. Palaeontographica Abteilung A. 2009; 287:123- 220.

41. Jiang D-Y, Rieppel O, Fraser NC, Motani R, Hao W-C, Tintori A, Sun Y-L, Sun Z-Y. Skeletal anatomy of *Macrocnemus fuyuanensis* Li et al., 2007, based on a new specimen from the latest Ladinian/earliest Carnian of the Middle/Late Triassic of Southwestern China. Journal of Vertebrate Paleontology. 2011; 31, 1230–1237.

42. Renesto S, Dalla Vechia FM. A revision of *Langobardisaurus rossii* Bizarrini and Muscio 1995 from the Late Triassic of Friuli (Italy). Rivista Italiana di Paleontologia e Stratigrafia. 2007; 113:191-201.

43. Rieppel O, Jiang D-Y, Fraser NC, Hao W-H, Motani R, Sun Y-L, Sun Z-Y. *Tanystropheus cf. T. longobardicus* from the early Late Triassic of Guizhou Province, southern China. Journal of Vertebrate Paleontology. 2010; 30:1082–1089.

44. Dilkes DW. The Early Triassic rhynchosaur *Mesosuchus browni* and the interrelationships of basal Archosauromorph Reptiles. The Royal Society. 1998; 353:501-541.

45. Sahney S, Benton MJ. Recovery from the most profound mass extinction of all time. Proc Biol Sci. 2008; 275:759–765.

46. Renesto S, Dalla Vecchia FM, Peters D. Morphological evidence for Bipedalism in the Late Triassic Prolacertiform Reptile *Langobardisaurus*. Senckenbergiana lethaea. 2002; 82:95-106.

47. Peters D. Description and interpretation of interphalangeal lines in tetrapods. Ichnos, Chur & New York. 2000; 7:11-41.

48. Renesto S, Avanzini M. Skin remains in a juvenile *Macrocnemus bassanii* Nopcsa (Reptilia, Prolacertiformes) from the Middle Triassic of northern Italy. Neues Jahrbuch für Geologie und Paläontologie Abhandlungen. 2002; 224, 31–48.

49. Jaquier VP, Scheier TM. Bone histology of the Middle Triassic long-necked reptiles *Tanystropheus* and *Macrocnemus* (Archosauromorpha, Protorosauria). Journal of Vertebrate Paleontology. 2017; 37.

50. Holz M, Souto-Ribeiro A. Taphonomy of the south-Brazilian Triassic vertebrates. Revista Brasileira de Geociências. 2000; 30:487–490.

Figure 1

Click here to access/download;Figure;Fig 1.tiff

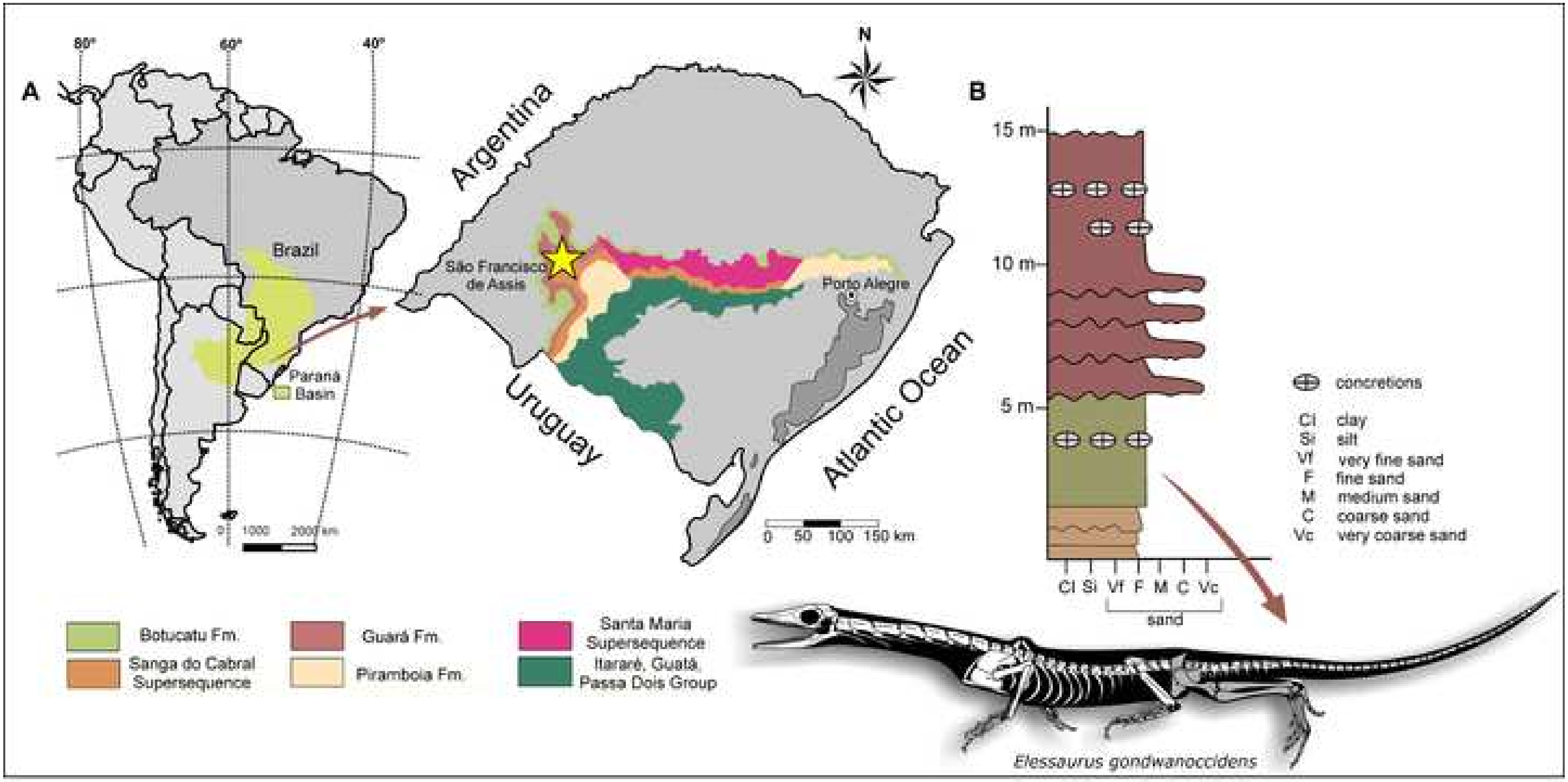

Figure 2

Click here to access/download;Figure;Fig 2.tiff

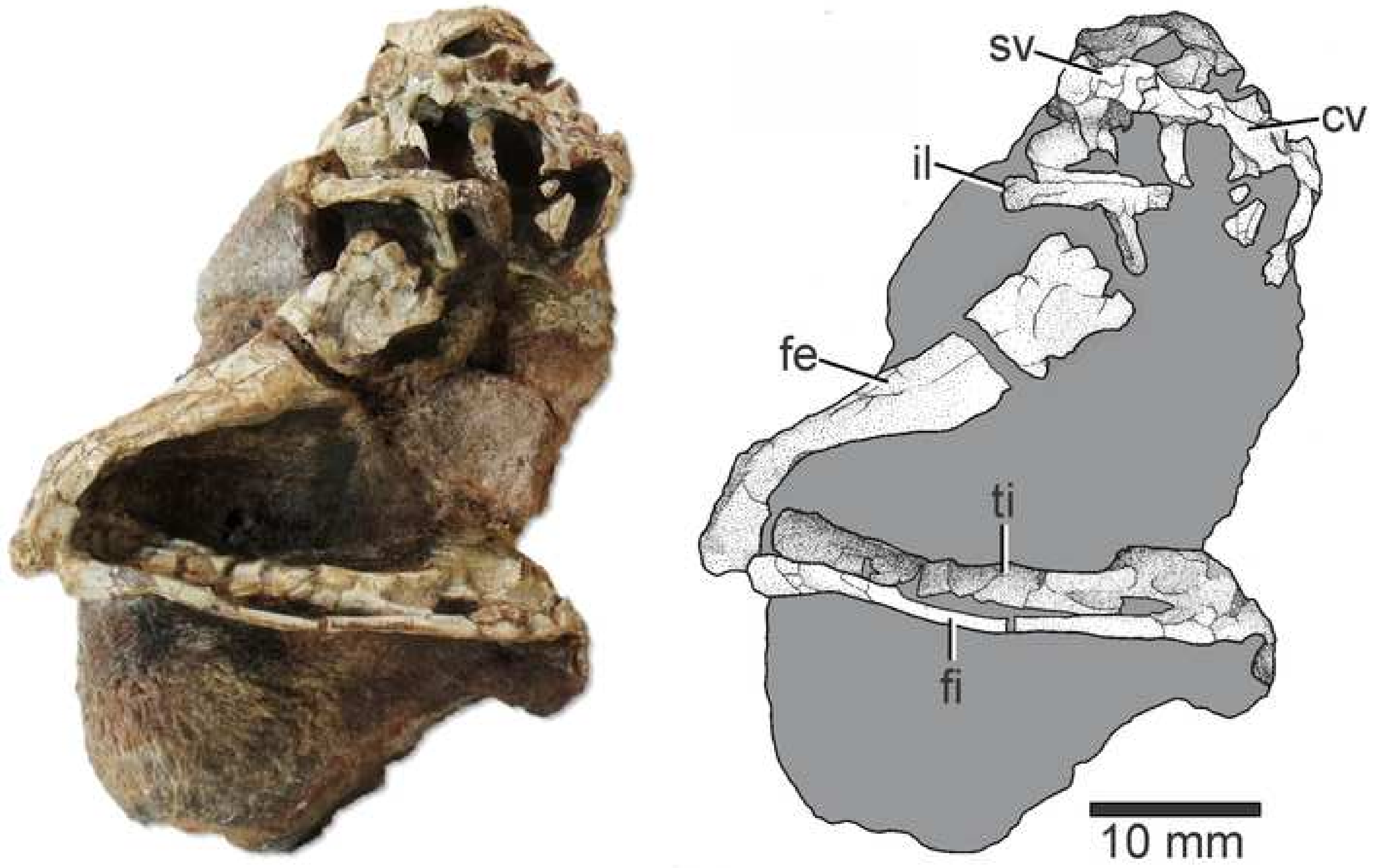

Figure 3

Click here to access/download;Figure;Fig 3.tiff

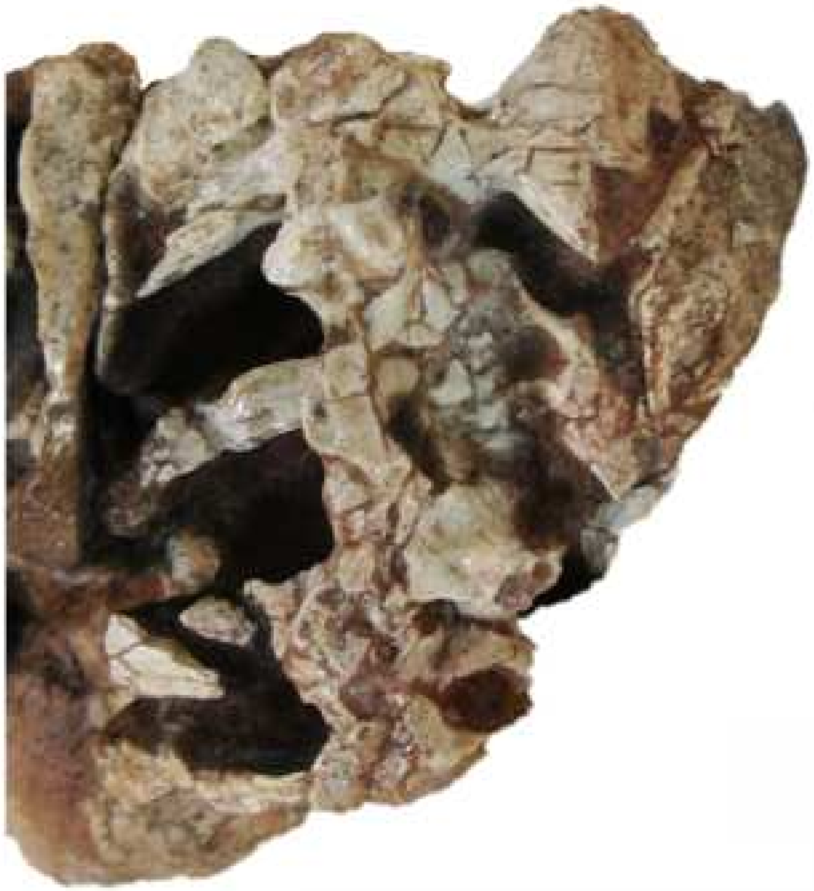
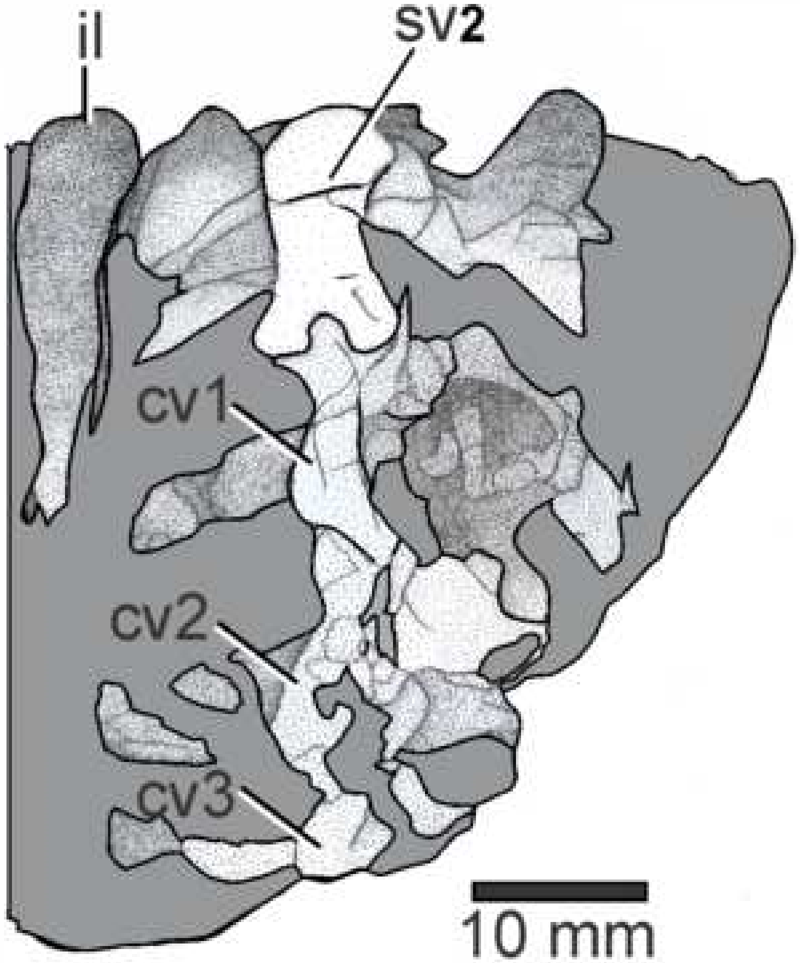

Figure 4 Click here to access/download;Figure;Fig 4.tif

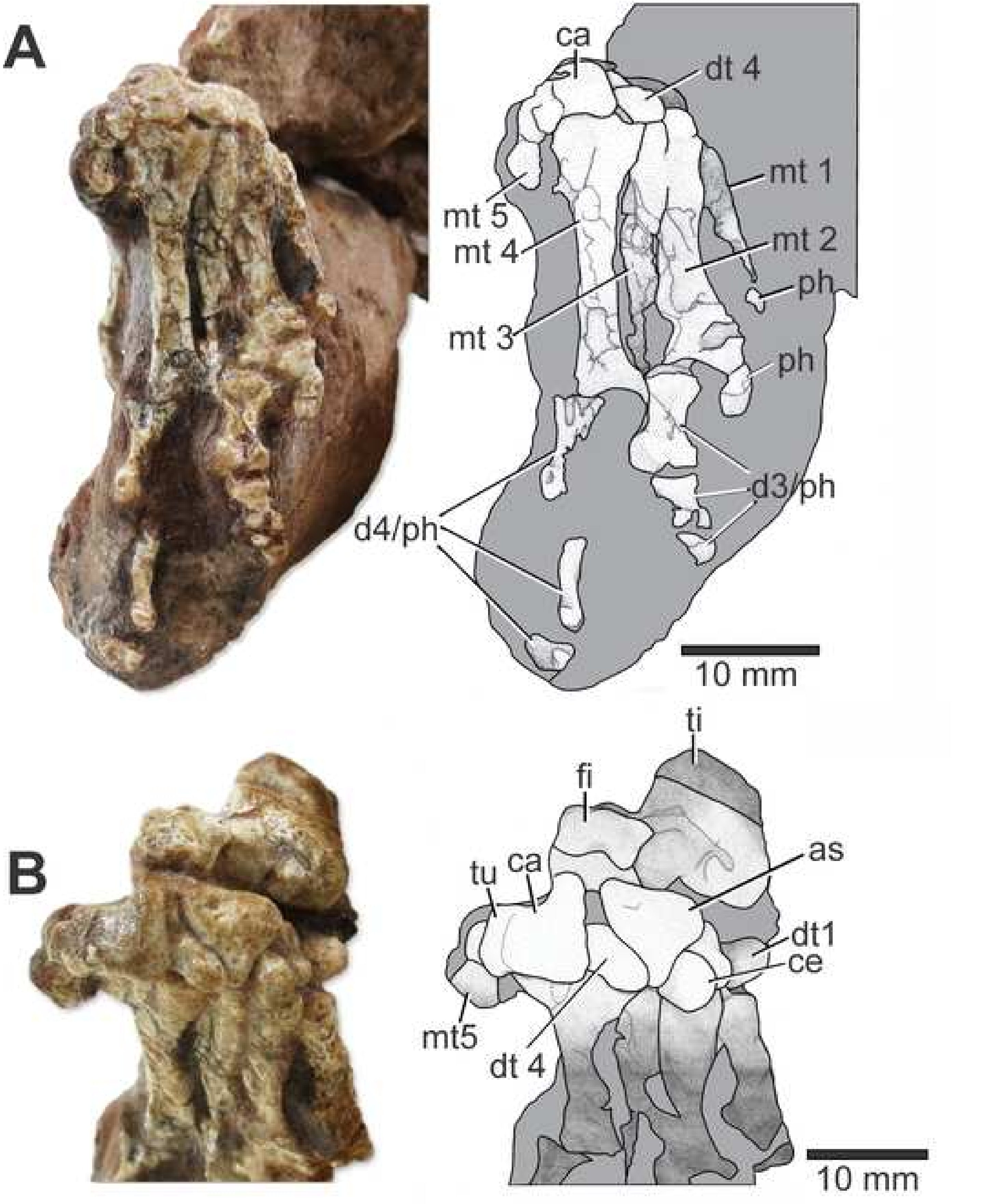

Figure 5

Click here to access/download;Figure;Fig 5.tiff

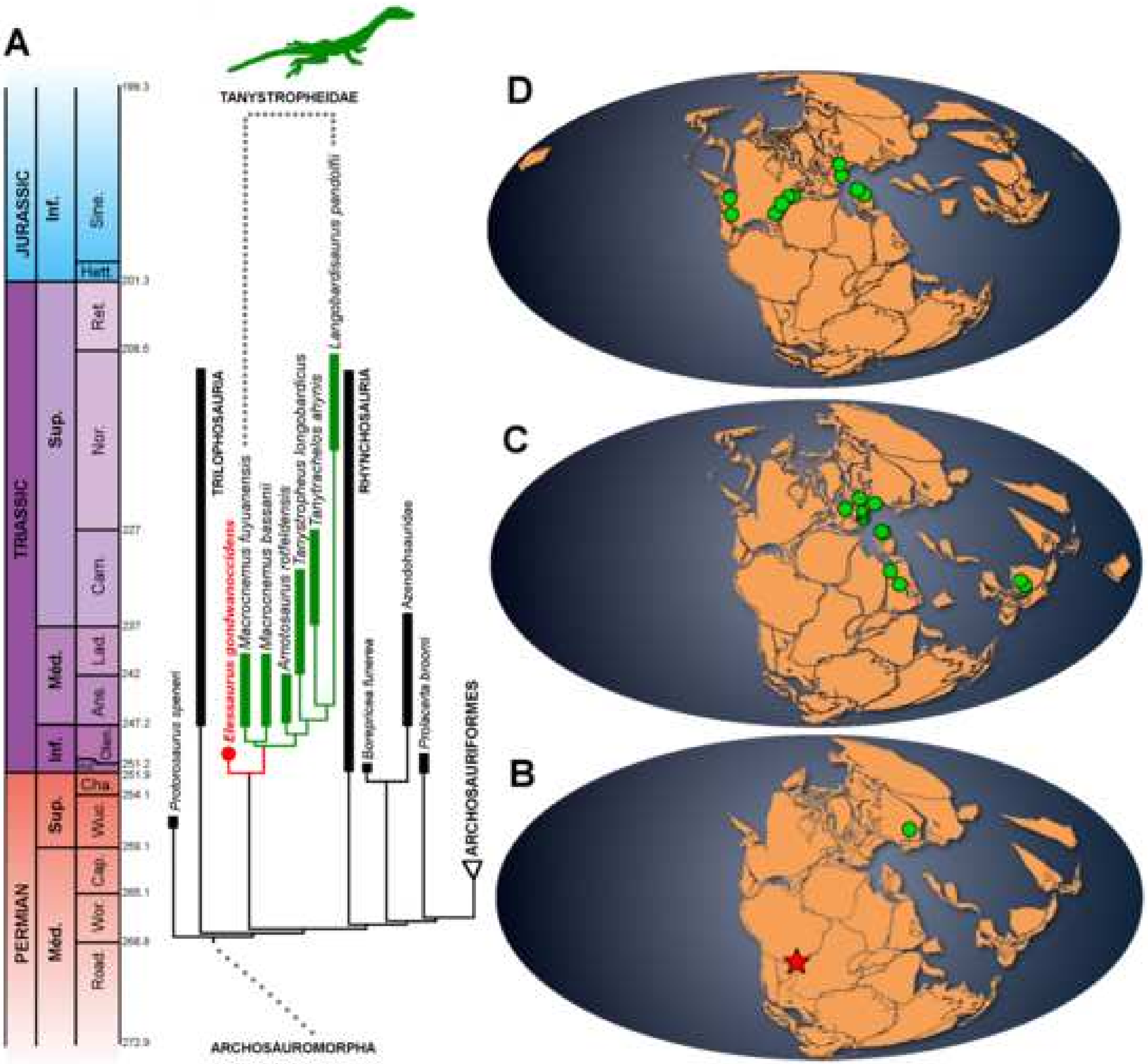

Click here to access/download
Supporting Information
supplementary_material.nex
