# Supplementary material for: A new archosauromorph from South America provides insights on the early diversification of tanystropheids
Source: PLoS One. 2020 Apr 8;15(4):e0230890. doi: 10.1371/journal.pone.0230890 (PMC7141609; doi:10.1371/journal.pone.0230890)
Supplement: Attachment — Submitted filename: Response to Reviewers.docx [file pone.0230890.s004.docx]

Dear Dr. Jörg Fröbisch

Academic Editor

PLOS ONE

We provide below a detailed point-by-point answer to reviewer’s comments of our manuscript PONE-D-19-19097 ‘*A new archosauromorph from South America provides insights on the early diversification of tanystropheids’.*

**Reviewer #1**

We thank the reviewer for its thorough review of the text, which substantially improved our MS presentation and content. In this respect, the reviewer’s corrections were fully adopted in the new version.

Our responses to the main concerns of Reviewer #1 are provided below:

- The revised version includes comparisons with both *Boreopricea* and *Dinocephalosaurus*;
- We did not exclude any taxon from the original dataset of Pritchard et al. (2015). In fact, the analysis of De-Oliveira et al. (2018) (mentioned by Reviewer #1) used a completely different data matrix – an updated version of Ezcurra (2016) dataset.
- *Jesairosaurus* and *Dinocephalosaurus* were included as OTUs in the dataset of Pritchard et al. (2015) and the results of this analysis is discussed and illustrated in the revised MS and supplementary material.
- The new taxon is the diagnosed by a combination of characters. The only potential autapomorphy (a lateral groove on the distal surface of the tibia) is probably taphonomic in origin, and this is discussed in the text.
- Some remarks were made about the potential correlation between *Elessaurus* and the tanystropheid vertebrae reported by De-Oliveira et al. (2018).
- The ecology of *Dinocephalosaurus* is briefly discussed.

**Reviewer #2**

The main issues raised by Dr. Martin Ezcurra concern the phylogenetic analysis methodology:

- We agree with Dr. Ezcurra in that our second analysis is superfluous and completely excluded it from the manuscript.
- Albeit we exploratively included *Elessaurus* in the dataset of Ezcurra (2016), we choose not to depict it in the revised version for the reasons below:

1. The original dataset of Ezcurra (2016) has a low tanystropheid representativity, as this study was designed to test the relationships among main archosauromorph clades, with emphasis on early archosauriforms. An updated version of the dataset was provided by Ezcurra & Butler (2018), which included score propositions for a number of tanystropheids that were absent from the original work. However, the *a posteriori* addition of new OTUs resulted in instability of the dataset as a whole, as the data matrix of Ezcurra & Butler (2018) was designed for morphological disparity analyses rather than for testing phylogenetic relationships. As such, we consider the data matrix of Pritchard et al. (2015) more suitable for our purposes.
2. Inclusion of *Elessaurus* in the dataset of Ezcurra (2016) recovered an apparently aberrant topology, in which the new taxon is recovered in a position we judge incompatible with its morphology. Tracking the character states that would sustain a derived position for *Elessaurus* in this explorative analysis, we noted the decisive influence of the presence and morphology of a calcaneal tuber and the hook-shaped metatarsal V. Although in the dataset of Ezcurra (2016) these characters are recovered as synapomorphic for Crocopoda, non-sampled unambiguous tanystropheids can also bear these features in a derived condition (e.g. *Tanytrachelos*, see Pritchard et al., 2015). As we thoroughly discuss in our manuscript, although the new specimen being incomplete, the morphology of *Elessaurus* is only fully compatible with tanystropheid-like archosauromorphs, and this relationship is in agreement with the dataset of Pritchard et al. (2015), this later including a broader sample of tanystropheids (and also *Tanytrachelos*). We, thus, prefer to stick with Pritchard’s dataset in combination with our comparative anatomical analyses.

We thank for the time dispensed in editing and reviewing our work.

Yours sincerely

Tiane De-Oliveira

Universidade Federal do Pampa

Universidade Federal de Santa Maria
