# Supplementary material for: A new archosauromorph from South America provides insights on the early diversification of tanystropheids
Source: PLoS One. 2020 Apr 8;15(4):e0230890. doi: 10.1371/journal.pone.0230890 (PMC7141609; doi:10.1371/journal.pone.0230890)
Supplement: Attachment — Submitted filename: Response to Reviewers.docx [file pone.0230890.s005.docx]

Dear Dr. Jörg Fröbisch

Academic Editor

PLOS ONE

We provide below a detailed point-by-point answer to reviewer’s comments of our manuscript PONE-D-19-19097 ‘*A new archosauromorph from South America provides insights on the early diversification of tanystropheids’.*

**Reviewer #1**

We thank the reviewer for its thorough review of the text, which were included in the new version. The suggestions substantially improved our manuscript presentation and content.

Our responses to the main concerns of Reviewer #1 are provided below:

- According to the suggestion of Reviewer #1, we prefer to make our phylogenetic position of *E. gondwanoccidens* less emphatic, as an enigmatic archosauromorph closely related to tanystropheids, which is in accordance to morphology and the recovered phylogenetic position in the dataset of Pritchard et al. (2018). At the end of the current work, *Elessaurus* is treated as a sister-taxon of Tanystropheidae. This result is in accordance to the occurrence of fossils related to tanystropheids in Sanga do Cabral Formation (De-Oliveira et al. 2018).
- *Elessaurus gondwanoccidens* was thoroughly codified in the matrixes of Ezcurra (2016), Ezcurra & Butler (2018) and Pritchard et al. (2018). We also included an additional analysis with the *a posteriori* inclusion of *Dinocephalosaurus orientalis* and *Jesairosaurus lehmani*, also in accordance with the reviewer’s requests.
- The dataset of Liu et al (2017) has the same tanystropheid representativity to what is displayed by our second dataset (with the addition of *Dinocephalosaurus* and *Jesairosaurus* to the matrix of Pritchard et al. 2018). As such, we believe that the effort of scoring *Elessaurus* to Liu et al (2017) dataset wouldn’t add to the resolution of *Elessaurus* relationships. We should also note that the dataset of Liu et al. (2017) is a composite of three previous data matrixes: Benton et al. (1997), Jalil (1997) and Dilkes (1998). Despite these being seminal works dealing with archosauromorph phylogeny, much has been done after these three contributions, and the datasets of Pritchard et al. (2018) and Ezcurra (2016) are much more reliable and up-to-date than previous works.
- As mentioned earlier, the original dataset of Ezcurra (2016) has a low tanystropheid representativity. The updated version of the dataset provided by Ezcurra & Butler (2018), resulted in instability of the dataset as a whole, as the data matrix of Ezcurra & Butler (2018) was designed for morphological disparity analyses rather than for testing phylogenetic relationships. As such, we consider the data matrix of Pritchard et al. (2018) more suitable for our purposes.
- As we thoroughly discuss in our manuscript, although the new specimen is incomplete, the morphology of *Elessaurus* is only fully compatible with tanystropheid-like archosauromorphs, and this relationship is in agreement with the dataset of Pritchard et al. (2018), this later including a broader sample of tanystropheids (and also *Tanytrachelos*). Again, we should highlight that the tanystropheid affinities of *Elessaurus* is in agreement with the Sanga do Cabral Fm fauna (De-Oliveira et al. 2018).

**Reviewer #2**

The main issues raised by Dr. Martin Ezcurra concern the phylogenetic analysis methodology:

- The changes in the results of phylogenetic analyses have been added to the text, as well, the discussion of the alternative positions that the new taxon adopt among the MPTs of the second analysis.
- We agree that other phylogenetic analyses are not completely necessary for the publication of the manuscript, and it is something that could be explored in future contributions.

We thank for the time dispensed in editing and reviewing our work.

Yours sincerely

Tiane De-Oliveira

Universidade Federal do Pampa

Universidade Federal de Santa Maria
