# Supplementary material for: A new archosauromorph from South America provides insights on the early diversification of tanystropheids
Source: PLoS One. 2020 Apr 8;15(4):e0230890. doi: 10.1371/journal.pone.0230890 (PMC7141609; doi:10.1371/journal.pone.0230890)
Supplement: Attachment — Submitted filename: Response to reviewers.docx [file pone.0230890.s007.docx]

Response to reviewers

All changes were made in accordance to the editor’s request.
